# Mutation- and Transcription-Driven Omic Burden of Daptomycin/Dalbavancin-R and Glycopeptide-RS Fitness Costs in High-Risk MRSA: A Nexus in Antimicrobial Resistance Mechanisms—Genome Proneness—Compensatory Adaptations

**DOI:** 10.3390/antibiotics14050465

**Published:** 2025-05-02

**Authors:** Eleonora Chines, Gaia Vertillo Aluisio, Maria Lina Mezzatesta, Maria Santagati, Viviana Cafiso

**Affiliations:** 1 Department of Biomedical and Biotechnological Sciences, University of Catania, 95123 Catania, Italy; eleonora.chines01@universitadipavia.it (E.C.); gaia.vertilloaluisio@unict.it (G.V.A.); mezzate@unict.it (M.L.M.); m.santagati@unict.it (M.S.); 2 Department of Public Health, Experimental, and Forensic Medicine, University of Pavia, 27100 Pavia, Italy

**Keywords:** fitness cost omic basis, essential genes, virulomics, DAP/GLY MOA-related AMR mechanisms, genome proneness, compensatory mutations, transcription-shifts

## Abstract

Background: In *Staphylococcus aureus*, antimicrobial resistance (AMR) imposes significant fitness costs (FCs), including reduced growth rate, interbacterial competitiveness, and virulence. However, the FC molecular basis remains poorly understood. This study investigated the FC omic basis and compensatory adaptations in high-risk HA-, LA-, and CA-MRSA, acquiring mono- or cross-resistance to second-line daptomycin (DAP) and dalbavancin (DAL), as well as reduced susceptibility (RS) to first-line glycopeptides, i.e., vancomycin and teicoplanin (GLYs, i.e., VAN, TEC), related to the specific mechanism of action (MOA)-related AMR-mechanisms and genomic backgrounds, paying increasing FCs. Methods: The FC omic basis associated with mono- or cross- DAP-/DAL-R and GLY-RS were investigated by integrated omics. This study focused on core-genome essential (EG) and accessory virulence gene (VG) SNPomics and transcriptomics by Illumina MiSeq whole-genome sequencing, RNA-seq, and bioinformatic analysis. Results: Moderate impact nsSNPs were identified in EGs related to vital cellular functions and VGs. Comparative EG transcriptomics revealed differential expressions and key dysregulations—via asRNAs—prevalently affecting the protein synthesis and cell-envelope EG clusters, as well as the VG cluster. Conclusions: Our data, firstly, underlined the EG and VG mutation- and transcription-driven omic-based FC burden and the compensatory adaptations associated with the emergence of mono-DAP-R, cross-DAP-R/hGISA, and DAP-R/DAL-R/GISA, linked to specific MOA-related AMR-mechanisms and genomic backgrounds in high-risk HA-, LA-, and CA-MRSA.

## 1. Introduction

Antimicrobial resistance (AMR) in Staphylococcus aureus poses a significant challenge to public health. This often confers fitness costs (FCs) that can reduce bacterial growth, competitiveness, or virulence in the absence of antimicrobials [1]. FCs can arise from molecular mechanisms such as target site modifications, efflux pump overexpression, and the metabolic burden of resistance gene maintenance.

Genomic plasticity can play a crucial role in balancing antimicrobial resistance FCs. The ability to acquire, lose, or modify genetic elements—such as plasmids, transposons, and mobile resistance genes—and compensatory mutations allows adaptations to the antimicrobial selective pressure, minimizing fitness trade-off [2]. 

Moreover, the genetic diversity of AMR mechanisms can influence the emergence and/or spread rate of specific AMRs. A diverse array of genes may play a role in resistance by chromosomal mutations, with some changes being more costly than others [3,4,5]. Mutations or the differential gene expression of essential genes (EGs)—responsible for vital cellular functions such as transcription, translation, or cell-wall biogenesis—can lead to FCs, impacting the ability to survive and compete in different environments such as the human host [6,7].

The occurrence of FC mutations is subject to the influence of diverse factors, including the genomic background, biochemical effects, and ecological context. Some mutations may result in a complete loss of function and severe FCs, whilst others may have more subtle effects or even confer a fitness advantage in certain conditions [8].

Since resistant bacteria are not always able to balance acquired AMR with their FCs [9], the level of FCs has a great influence on the rate at which resistance develops, the stability of the resistance, and the rate at which resistance can decline if antibiotic use is reduced [3,4,10].

Lastly, AMR acquisition can also affect the virulence, i.e., the ability of bacteria to invade a host and cause damage. Even though virulence factors are not essential for the growth and survival of bacteria in the absence of other competing factors [11], they represent the first pathway to be affected to balance antimicrobial resistance FC [12,13].

Three genetically distinct MRSA pools are recognized i.e., hospital-associated MRSA (HA-MRSA), community-acquired MRSA (CA-MRSA), and livestock-associated MRSA (LA-MRSA) [14]. Each MRSA clone emerged through genome remodeling due to the acquisition of resistance, virulence, and host adaptation genes, coupled with selective pressures such as antimicrobial usage. Notably, CA-MRSA is typically more virulent than HA-MRSA and, conversely, shows higher levels of antimicrobial resistance [12]. Conversely, LA-MRSA strains are characterized by an accessory genome lacking human-associated virulence and adhesion determinants, and they form a distinct homogeneous lineage from HA- and CA-MRSA strains [15].

Glycopeptides (GLYs), including vancomycin (VAN) and teicoplanin (TEC), represent the first-line treatment of MRSA serious infections [16,17]. On the contrary, daptomycin (DAP), dalbavancin (DAL), linezolid, tigecycline, and ceftaroline are second-line options [18]. 

However, MRSA widespread led to increased reliance on glycopeptides, leading to the emergence of strains with reduced susceptibility or resistance. This includes the heterogeneous glycopeptide-intermediate *S. aureus* (hGISA) and glycopeptide-intermediate *S. aureus* (GISA) phenotypes, which display adaptive changes such as a thickened cell-wall and altered metabolism, thus reducing glycopeptide efficacy. In this context, dalbavancin is a second-generation lipoglycopeptide antimicrobial, with potent activity towards MRSA and GISA, by binding to the D-alanyl-D-alanine terminus of peptidoglycan precursors, inhibiting cell wall synthesis and leading to bacterial cell death [19]. Dalbavancin exhibits enhanced activity compared to vancomycin and teicoplanin due to its stronger binding affinity to the D-alanyl-D-alanine target site of peptidoglycan precursors. This strengthened adherence increases its potency, allowing for prolonged bacterial inhibition at lower concentrations [18]. 

Daptomycin acts by binding to the bacterial cell membrane, causing rapid depolarization, loss of membrane potential, and the inhibition of protein, DNA, and RNA synthesis, ultimately leading to bacterial cell death; however, resistance can arise through mutations in membrane charge, alterations in membrane fluidity, or the upregulation of certain regulatory pathways that reduce daptomycin binding or function [20], leading to a positively charged drug repulsion mechanism [21].

These resistance mechanisms complicate MRSA infection treatment, necessitating careful antimicrobial stewardship and the development of novel therapeutic strategies [22].

Recently, we provided new insights into the FC-burden associated with the stepwise acquisition of reduced susceptibility to DAP and GLYs in high-risk MRSA [13]. The changes in growth performance, competitiveness, and virulence highlighted the trade-off that the bacteria face when acquiring resistance [13]. However, the molecular mechanisms underlying these fitness costs remain unexplored.

On this basis, our task was to investigate the DAP/DAL/GLY resistance-mechanisms, genome-proneness, and compensatory-adaptations in essential (EG) and virulence gene (VG) clusters in three isogenic high-risk HA-, LA-, CA-MRSA strain pairs that developed resistance, under mono or combined DAP/GLY therapy, paying increasing FCs.

Understanding these mechanisms can provide new insights to predict resistance evolution and strategies to counteract resistance development in community and clinical settings.

## 2. Results

We investigated three isogenic high-risk MRSA strain pairs: ST5 N315 (1-R vs. 1-S), ST398 (2-R vs. 2-S), and ST1 MW2 (3-R vs. 3-S), which are the prototypes of the most prevalent MRSA clones developing mono DAP-/DAL-R or hGISA/GISA cross-resistance, and paying low or high FCs (Table 1) [13]. 

In ST5 N315 DAP-R GSSA (1-R vs. 1-S), DAP-R onset FCs determined a very low in vivo FC burden due to a poor reduction in growth performance, competitiveness, and virulence (Table 1) [13].

In ST398 DAP-R hGISA (2-R vs. 2-S), DAP-R hGISA cross-resistance FCs determined a low in vivo burden leading to a medium slow growth, a high competitiveness, and maintaining a low virulence (Table 1) [13].

In ST1 MW2 DAP-R DAL-R GISA (3-R vs. 3-S), DAP-R DAL-R GISA cross-resistance FCs determined a high in vivo FC burden, which led to a simultaneous decrease in growth performance, competitiveness, and a huge reduction in virulence (Table 1) [13].

A focus on SNPomics and transcriptomics in essential (EG) and virulence gene (VG) clusters was performed to find out the DAP/DAL/GLY Mechanism of Action (MOA)-related resistance mechanisms, the omic basis, i.e., the “FC-compensatory” genomic proneness and mutational and transcriptional signatures.

### 2.1. DAP-R/DAL_R/GLY-R MOA-Related Resistance Mechanisms: nsSNPs and Resistance-Related Differentially Expressed Genes

All MOA resistance mechanisms related to DAP-R and DAL/GLY-R MOA in ST5 N315 DAP-R GSSA (1-R vs. 1-S), ST398 DAP-R hGISA (2-R vs. 2-S), and ST1 MW2 DAP-R DAL-R GISA (3-R vs. 3-S) are shown in Table 2.

In detail, SNPomics identified moderate-impact non-synonymous single-nucleotide polymorphisms (MI-nsSNPs) in fmtC/mprF (Phosphatidylglycerol L-lysyltransferase), a gene implicated in DAP-R via a positive net-charge repulsion mechanism. These mutations resulted in T345I (1-R), S295L in 2-R, and T345A (3-R) FmtC amino acid (AA) substitutions, leading to a bioinformatically predicted decreased protein stability, and thus, functionality, as indicated by the negative ΔΔG value change of −0.86 (1-R vs. 1-S), −0.52 (2-R vs. 2-S), and −1.62 (3-R vs. 3-S), respectively (Table S1). 

Comparative transcriptomics revealed tarF/L (teichoic acid biosynthesis and modification), dltA/C (peptidoglycan biosynthesis), and pgsA (phospholipid biosynthesis) over-expression, associated with mono-DAP-R, in 1-R; murA/I (peptidoglycan biosynthesis) related to hGISA and tarL over-expression to DAP-R, in 2-R; dltA and mprF (cell-envelope modification) over-expression related to DAP-R and murF over-expression coupled with an alr and ddl under-expression related to GISA, in 3-R (Table S2).

### 2.2. Omic FC-Related Burden in Acquiring Mono/Cross-Resistance to DAP/DAL and GLYs in High-Risk MRSA

Comparative nsSNPomics between R-strains and their S-parents was investigated to identify nsSNPs in EG and VG clusters (Table S1) to define the mutation-driven FC burden. Similarly, the transcription-driven FC burden was assessed using comparative transcriptomics, highlighting differential expressions in VG and EG genes, including mRNAs and antisense small RNAs (asRNAs). 

Affected EG and VG clusters, along with their subsets, were reported in decreasing the under-expression order for coding-targets and decreasing the amount for asRNAs by category with under-expressed (↓UE) and over-expressed (↑OE) genes. 

An overview of comparative transcriptomics is shown in Table S2.

#### 2.2.1. ST5 N315 DAP-R HA-GSSA vs. DAP-S HA-GSSA FC Omic Burden

##### nsSNPs in EG and VG clusters

Comparative EG SNPomics between ST5 N315 DAP-R HA-MRSA 1-R and DAP-S HA-MRSA 1-S identified two EG nsSNPs. One MI-nsSNP was found in the cell envelope charge-EG *mprF* (T345I), as described above, associated with mono-DAP-R, and a wild-type revertant SNP was found in the peptidoglycan biosynthesis EG *fmhB* (K68T) (Table S1).

##### EG and VG Differential Expressed Gene-Clusters

Comparative transcriptomics between ST5 N315 DAP-R HA-MRSA 1-R and DAP-S HA-MRSA 1-S revealed statistically significant differential expressions (q < 0.01) as follows (Table S2):

Coding-Targets:

(1) Protein Synthesis EGs: -24 mRNAs (7↓-17↑)-: Ribosomal Protein (4↓-9↑): UE in rplS, rpsS, rplD, rplC and OE in rpsR, rplJ, rpsD, rpsM, rpsE, rplN, rplB, rplY, rplL; tRNA synthetase (1↓-4↑): UE in thrS and OE in argS, pheT, leuS, gatB; Protein-Folding (1↓-2↑): UE in groEL and OE in dnaK, grpE; Translocation Factor (1↓-2↑): UE in efp, and OE in tsf, infB.

(2) Unknown/Other Function EGs: -13 mRNAs (7↓-6↑)-: UE in ppaC, plsY, pmtD, ybeY, SA0003, SA0878, SA1445 and OE in pdhA, ltaS, dmpI, SA1293, SA1501, SA1375.

(3) Cell-Envelope EGs: -15 mRNAs (4↓-11↑)-: Teichoic Acid Modification (4↑): OE in tarF, tarL, dltA, dltC; Peptidoglycan Biosynthesis (2↓-1↑): UE in murT, femX and OE in pbp2. Cell Division (2↓-2↑): UE in ftsZ, gidA and OE in ezrA, noc; Lipid Biosynthesis (2↑): OE in accC, accD; Phospholipid Biosynthesis (1↑): OE in pgsA; Cell envelope—other (1↑): OE in ebpS.

(4) Carbon Metabolism EGs: -6 mRNAs (4↓-2↑)-: Glycolysis (2↓-2↑): UE in prs, eno and OE in pgk, tkt; Pentose phosphate (1↓): UE in gnd; Intermediary metabolism (1↓): UE in sucC.

(5) Cofactor Metabolism EGs: -7 mRNAs (3↓-4↑)-: FE-sulfate cluster (1↓-2↑): UE in sufU and OE in sufS, fer; NAD biosynthesis (1↓-1↑): UE in nadD and OE in pncB; acetyl-CoA/COA (1↑): OE in coaBC; Folate (1↓): UE in folK.

(6) Respiratory Pathway EGs: -4 mRNAs (2↓-2↑)-: Isoprenoid/mevalonate biosynthesis (1↓-2↑): UE in mvaS and OE in SA0342, mvaD; Thioredoxin (1↓): UE in trxA.

(7) Nucleotide Metabolism EGs: -4 mRNAs (2↓-2↑)-: Purine and pyrimidine metabolism (2↓-2↑): UE in gmk, nrdE and OE in adk, relA.

(8) Virulence Cluster: -13 mRNAs (1↓-12↑)-: Adhesin (5↑): OE in sdrC, ebpS, fnbA, icaC, manP/cna; Immune Evasion (4↑): OE in adsA, capB, capG, capL; Exoenzyme (1↓-2↑): UE in lip and OE in geh, splA; Secretion Systems (1↑): OE in esxA.

(9) RNA Metabolism EGs: -5 mRNAs (5↑)-: Basic transcription machinery (3↑): OE in rpoC, sigA, rpoA; RNA regulation (2↑): OE in spxA, msrR.

(10) DNA Metabolism EGs: -4 mRNAs (4↑)-: DNA replication (4↑): OE in 1 ssb, dnaG, 1 holA, ligA.

Regulatory-Targets:

(1) Protein Synthesis EGs: -14 asRNAs (3↓-11↑)-: tRNA synthetase (2↓-4↑): UE in ileS, gatC and OE in 2 lysS, trpS, alaS, glyS; Ribosomal Protein (5↑): OE in rpsF, 2 rpsR, rplA, rpmB, 2 rplY; Protein-Folding (1↑): OE in 2 dnaK. Protein-Translocation (1↓-1↑): UE in secY and OE in yidC.

(2) Cell-Envelope EGs: -14 asRNAs (2↓-12↑)-: Peptidoglycan Biosynthesis (4↑): OE in pbp2, murT, ddl, murB; Teichoic Acid Modification (3↑): OE in tagH, llm, dltA; Cell-wall amino sugar (1↓-2↑): UE in 2 glmS and OE in 3 glmS, 2 glmU; Cell Division (1↓-1↑): UE in gpsB and OE in sepF; Lipid Biosynthesis (1↑): OE in accC; Cell Envelope modification (1↑): OE in fmtC;

(3) Unknown/Other Function EGs: -7 asRNAs (2↓-5↑)-. UE in ppaC, ssaA and OE in plsY, SA1426, SA0816, SA1445, SA1425.

(4) RNA Metabolism EGs: -7 asRNAs (2↓-5↑)-: RNA regulation (2↓-2↑): UE in greA, spxA and OE in fur, 2lexA. Basic transcription machinery (2↑): OE in rpoB, rpoC; RNA modification (1↑): OE in 2 rnjA.

(5) Virulence Cluster: -6 asRNAs (2↓-4↑)-: Adhesin (1↓-4↑): UE in sdrC and OE in sdrC, 3clfB, clfA, icaA-icaD; Secretion Systems (1↓): UE in esaD.

(6) Carbon Metabolism EGs: -4 asRNAs (1↓-3↑)-: Glycolysis (1↓-2↑): UE in fbaA and OE in pgi, fbaA; Pentose phosphate (1↑): OE in zwf.

(7) Cofactor Metabolism EGs: -3 asRNAs (1↓-2↑): FE-sulphate cluster (1↓-2↑): UE in sufB and OE in sufC, sufD.

(8) Respiratory Pathway EGs: -2 asRNAs (1↓-1↑)-: Isoprenoid/mevalonate biosynthesis (1↓-1↑): UE in fni and OE in mvaS.

(9) DNA Metabolism EGs: -2 asRNAs (2↑)-: DNA replication (2↑): OE in ssb, 2 holA.

**Figure 1:**
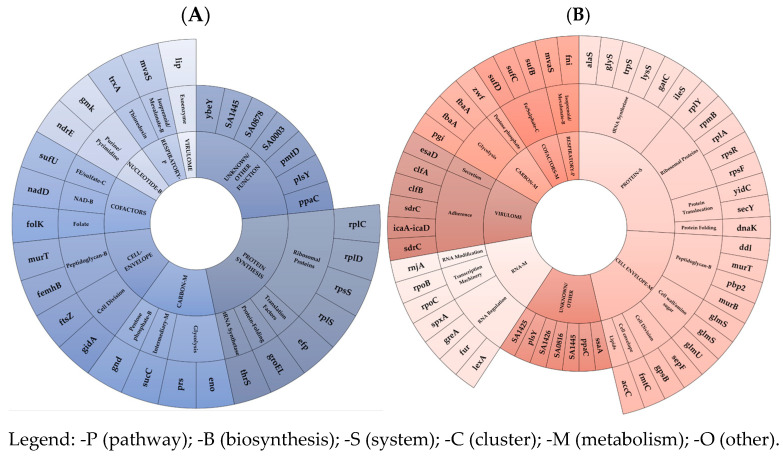
Under-expressed mRNAs (**A**) and dysregulated asRNAs (**B**) in EG and VG clusters/subsets in ST5 N315 1-R vs. 1-S.

Under-expressed mRNAs (A) and dysregulated asRNAs (B) in EG and VG clusters/subsets in ST5 N315 1-R vs. 1-S were shown in Figure 1.

#### 2.2.2. ST398 DAP-R hGISA vs. DAP-S GSSA FC Omic Burden

##### nsSNPs in EG and VG clusters

In ST398 DAP-R hGISA 2-R vs. DAP-S GSSA 2-S, comparative SNPomics evidenced four MI-SNPs in EGs. In detail, three MI-nsSNPs were found, i.e., in the cell-envelope charge-EG mprF (S295L), in basic transcription machinery EG rpoC (V496A) (Transcription), in unknown/other EG SAPIG1881 exported protein (Y66N) (Table S1), and one wild-type revertant MI-nsSNP was found in the basic transcription machinery EG rpoB associated with RIF-R and transcription (N471A) (Table S1).

Comparative VG SNPomics revealed one MI-nsSNP in ebh (S6725I) (cell-wall-associated fibronectin-binding protein) (Table S1).

##### EG and VG Differential Expressed Gene-Clusters

In ST398 DAP-R hGISA 2-R vs. DAP-S GSSA 2-S, comparative transcriptomics highlighted statistically significant differential expressions (q < 0.01) as follows (Table S2):

Coding-Targets:

(1) Protein Synthesis EGs: -14 mRNAs (7↓-7↑)-: tRNA Synthetase (2↓-2↑): UE in serS, gatB and OE in leuS, gatA; Ribosomal Protein (2↓-2↑): UE in rpmF, rpmA and OE in rpsC, rplT; Protein-Folding (2↓-1↑): UE in grpE, groEL and OE in dnaJ; Protein-Translocation (1↓-): UE in spsB; Translation-Factors (2↑): OE in tsf, efp.

(2) Unknown/Other Function EGs: 6-mRNA (4↓-2↑)-: UE in ppaC, rplGA, SAPIG1610, SAPIG1681 and OE in SAPIG1156, dmpI.

(3) Cofactor Metabolism EGs: -5 mRNAs (4↓-1↑)-: FE-Sulphate Cluster (2↓-1↑): UE in sufC, iscS and OE in sufB; Folate (2↓): UE in folE2, folC.

(4) DNA Metabolism EGs: -5 mRNAs (3↓-2↑)-: DNA Replication (2↓-1↑): UE in dnaG, holA and OE in dnaI; DNA Packaging and Segregation (1↓-1↑): UE in hu and OE in SAPIG1680.

(5) Virulence Cluster: -4 mRNAs (3↓-1↑)-: Immune Evasion (1↓-1↑): UE in SAPIG0171 and OE in SAPIG0168; Exoenzyme (1↓): UE SAPIG2721; Exotoxin (1↓): UE SAPIG1158.

(6) RNA Metabolism EGs: -4 mRNAs (3↓-1↑)-: RNA Modification (3↓): UE in rnjA, rimM, trmD; Basic Transcription Machinery (1↑): OE in sigA. 

(7) Cell-Envelope Metabolism EGs: -7 mRNAs (2↓-5↑)-: Peptidoglycan Biosynthesis (1↓-2↑): UE in lytH OE in murI, murA; Teichoic Acid Modification (1↑): OE in tarL; Lipid Biosynthesis (2↑): OE in acpP, acpS; Cell-Wall Amino Sugar (1↓): UE in glmM.

(8) Respiratory Pathway EGs: -4 mRNAs (1↓-3↑)-: Isoprenoid/Mevalonate Biosynthesis (1↓-3↑): UE in mvaK1 and OE in mvaA, mvaS, SAPIG0434. 

(9) Carbon Metabolism EGs: -3 mRNAs (1↓-2↑)-: Glycolysis (1↓-1↑): UE in pgi and OE in prsA; Regulation (1↑): OE in ptsH.

Regulatory Targets:

(1) RNA Metabolism EGs -4 asRNAs: (3↓-1↑)-: RNA Modification (1↓): UE in 2 rnjA; RNA Regulation (1↓-1↑): UE in fur and OE in spxA; Basic Transcription Machinery (1↓): UE in rpoB. 

(2) Protein Synthesis EGs: -4 asRNAs (1↓-3↑)-: Ribosomal Protein (1↓-1↑): UE in rpsD and OE in 2 rpsD. Translation Factor (2↑): OE in dtd, fusA.

(3) Virulence Cluster: 4 asRNA (1↓- 3↑): Adherence (1↓-2↑)-: UE in sdrC and OE in atl, sdrD; Immune evasion (1↑): OE in capC.

(4) DNA Metabolism EGs: -3 asRNAs (3↓)-: DNA Replication (3↓): UE in 2 ssb, holA, pcrA.

(5) Cofactor Metabolism EGs: -1 asRNAs (1↓)-: FE-Sulphate Cluster (1↓): UE in sufB. 

(6) Respiratory Pathway EGs: -1 asRNAs (1↓)-: Isoprenoid/Mevalonate Biosynthesis (1↓): UE in mvaS. 

(7) Unknown/Other Function EGs: -1 asRNAs (1↓)-: UE in ltaS.

(8) Cell-Envelope Metabolism EGs: -1 asRNAs (1↑)-: Cell Envelope Modification (1↑): OE in 3 fmtC.

**Figure 2:**
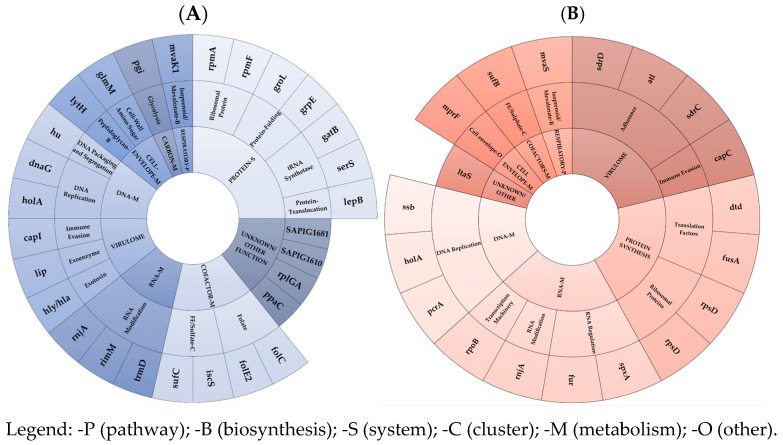
Under-expressed mRNAs (**A**) and dysregulated asRNAs (**B**) in EG and VG clusters and subsets in ST398 2-R vs. 2-S.

Under-expressed mRNA coding genes (**A**) and dysregulated asRNAs (**B**) in EG and VG clusters and subsets in ST398 2-R vs. 2-S were shown in Figure 2.

#### 2.2.3. ST1 MW2 DAP-R DAL-R CA-GISA vs. DAP-S DAL-S CA-GSSA FC Omic Burden

##### nsSNPs in EG and VG clusters

Comparative genomics between ST1 MW2 DAP-R DAL-R CA-GISA 3-R and DAP-S DAL-S CA-GSSA 3-S identified seven nsSNPs in EGs. Specifically, MI-nsSNPs were found in the basic transcription machinery EG rpoB (H481Y), in the cell-envelope charge-EG mprF (T345A). MI-SNPs, namely resistance-accessory unknown/other EG I186M GdpP (cyclic-di-AMP phosphodiesterase associated with β-lactam resistance and cross-resistance to glycopeptides) and I121N MurG (peptidoglycan Lipid-II biosynthesis-EG), were identified, which indirectly supports mechanisms of reduced glycopeptide susceptibility (Table S1). Three wildtype revertant MI-nsSNPs were identified in the following EGs, namely the F74L TagH for teichoic acid biosynthesis, V329G GlnA for glutamine biosynthesis, and N177K RecU involved in DNA packaging (Table S1). 

The comparative virulome SNPomics identified four MI-nsSNPs, namely Y130H in Cap8H, V120G in Cap8K (capsular polysaccharide synthesis enzymes), T1313S in SdrD (serine-aspartate repeat adhesion protein), and V1768D in Ebh (cell-wall-associated fibronectin-binding protein), in addition to one D635E ClfB in wild-type revertant MI-nsSNP (adhesion clumping factor B) (Table S1).

##### EG and VG Differential Expressed Gene Clusters

Statistically significant differential expressions (q< 0.01) in the EG and VG coding genes and EG and VG asRNAs were recorded for ST1 MW2 DAP-R DAL-R CA-GISA 3-R vs. DAP-S DAL-S CA-GSSA 3-S, given as follows (Table S2):

Coding-Targets:

(1) Protein Synthesis EGs: -23 mRNAs (14↓-9↑)-: Ribosomal Protein (8↓-4↑): UE in rpsF, rpsB, rplU, rplM, rplF, rpsC, rplV, rpsS and OE in rpsG, rpmF, rpsU, rpsM; t-RNA Synthetase (3↓-2↑): UE in metS, lysS, thrS and OE in aspS, valS; Translation Factor (1↓-2↑): UE in dtd and OE in tsf, infB; Protein-Folding (1↓-1↑): UE in grpE and OE in dnaJ; Protein-Translocation (1↓): UE ftsY.

(2) Virulence Cluster: -16mRNAs (14↓-2↑)-: Exotoxin (5↓): UE in hla, hld, hlgB, hlgC, seh; Immune Evasion (2↓-2↑): UE in esaA, cap8P and OE in cap8N, sbi; Adhesins (4↓): UE in sdrC, sdrD, eap/map, atl; Exoenzyme (1↓): UE in splB; Biofilm (1↓): UE in icaA; Membrane acting Superantigen and Toxins (1↓): UE in spa.

(3) Unknown/Other Function EGs: -12 mRNAs (9↓-3↑)-: UE in MW1973, MW0014, MW0708, MW1975, MW2090, MW1106, MW1151, MW2218, MW1548 and OE in MW1240, MW0837, MW0902.

(4) Carbon Metabolism EGs: -7 mRNAs (6↓-1↑)-: Glycolysis (4↓-1↑): UE in pgk, tpiA, pgi, gapA and OE in pfkA; Pentose Phosphate (1↓): UE in gnd; Regulation (1↓): UE in ptsH.

(5) DNA Metabolism EGs: -9 mRNAs (4↓-5↑)-: DNA Replication (4↓-3↑): UE in holA, dnaI, dnaB, pcrA and OE in priA, polC, dnaG; DNA Packaging and Segregation (2↑): OE in gyrB, hu.

(6) Cofactor Metabolism EGs: -7 mRNAs (4↓-3↑)-: FE-Sulfate Cluster (3↑): OE in sufD, sufU, fer; Folate (2↓): UE in folB, folE2; NAD Biosynthesis (1↓): UE ppnK; SAM (1↓): UE metK.

(7) Cell-Envelope Metabolism EGs: -12 mRNAs (4↓-8↑)-: Peptidoglycan Biosynthesis (2↓-2↑): UE in murI, ddl and OE in murF, uppS; Teichoic Acid Modification (1↓-2↑): UE in dltD and OE in dltA, dltB; Lipids (3↑): OE in fabG, birA, accC; Cell Envelope Modification (1↑): OE in fmtC; Phospholipid Biosynthesis (1↓): UE in plsC;.

(8) RNA Metabolism EGs: -7 mRNAs (2↓-5↑)-: RNA Regulation (2↓-3↑): UE in vicR, vicK and OE in nusA, rnr, msrR; RNA Modification (2↑): OE in rnjB, trmE.

(9) Nucleotide Metabolism EGs: -2 mRNAs (1↓-1↑)-: Purine and Pyrimidine Biosynthesis (1↓-1↑): UE in pyrH and with OE in adk.

(10) Respiratory Pathway EGs: -3 mRNAs (3↑)-: Menaquinone biosynthesis (1↑): OE in menA Thioredoxin (2↑): OE in trxA and trxB.

Regulatory-Targets:

(1) Protein Synthesis EGs: -17 asRNAs (15↓-2↑)-: Ribosomal Protein (7↓-1↑): UE in rpmF, rpsD, rplO, rplN, rpmC, rpsS, 2 rpsB and OE in rpsL; t-RNA Synthetase (3↓-1↑): UE in glyS, alaS, thrS and OE in valS; Protein Folding (2↓): UE 2 dnaK, groEL; Translation Factor (2↓): UE 2 infB, smpB/ssrP; Protein Translocation (1↓): UE in secF.

(2) Unknown/Other Function EGs: -16 asRNAs (9↓-7↑)-: UE in obgE, engA, MW0544, MW0837, MW1104, MW1350, MW1727, MW1860, MW0681 and OE in MW1860, MW1240, MW0544, MW1009, MW1692, MW2353, MW1872.

(3) Virulence Cluster: -15 asRNAs (13↓- 2↑)-: Adhesins (5↓-1↑): UE in sdrD, 4 clfA, clfB, atl, cna and OE in 2 sdrH; Exoenzyme (3↓-1↑): UE in lip, sspA, splB and OE in splC; Immune Evasion (2↓): UE in cap8O, sbi; Exotoxin (1↓): UE in hlgA; Membrane (1↓): UE in 2 spa; Effector delivery system (1↓): UE in essC.

(4) Cell-Envelope Metabolism EGs: -9 asRNAs (7↓-2↑)-: Peptidoglycan Biosynthesis (3↓): UE in murF, 2 murT, ddl; Cell Envelope Modification (1↓): UE in 2 mprF; Lipid Biosynthesis (1↓): UE in fabZ; Cell Division (1↓-1↑): UE in ftsA and OE in ftsL; Cell-Wall Amino Sugar (1↓): UE in glmU; Teichoic acid biosynthesis (1↑): OE in dltA.

(5) Cofactor Metabolism EGs: -9 asRNAs (7↓-2↑)-: Folate (2↓-1↑): UE in dfrA, 2 folC and OE in folC; FE-Sulfate Cluster (2↓-1↑): UE sufS, sufU and OE in sufS; NAD Biosynthesis (1↓): UE 2 nadD; SAM (1↓): UE metK; AcetylCoA/CoA (1↓): UE in coaA.

(6) DNA Metabolism EGs: -8 asRNAs (6↓-2↑)-: DNA Replication (3↓-1↑): UE in dnaA, 2 dnaN, 2 ssb and OE in ssb; DNA Packaging and Segregation (3↓-1↑): UE in 2 gyrA, gyrB, topA and OE in recU.

(7) RNA Metabolism EGs: -7 asRNAs (2↓-5↑)-: RNA Regulation (2↑): OE in fur, 2 msrR; Basic Transcription Machinery (1↓-2↑): UE in rpoB (RNA polymerase beta chain) and OE in 2 rpoB and rpoC; RNA Modification (1↓-1↑): UE in rnjA and OE in rnjA.

(8) Nucleotide Metabolism EGs: -4 asRNAs (2↓-2↑)-: Purine and Pyrimidine Biosynthesis (2↓-2↑): UE in gmk, nrdE and OE in nrdI and pyrG.

(9) Carbon Metabolism EGs: -3 asRNAs (3↓)-: Glycolysis (2↓): UE in pykA, fbaA, eno.

(10) Respiratory Pathway EGs: -2 asRNAs (1↓-1↑)-: Isoprenoid/mevalonate biosynthesis (1↓): UE in ispD/tarI; Menaquinone Biosynthesis (1↑): OE in menA.

**Figure 3:**
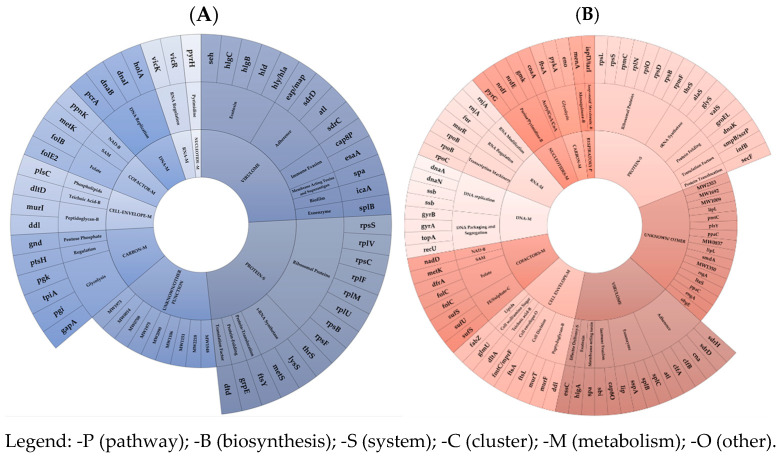
Under-expressed mRNAs (**A**) and dysregulated asRNAs (**B**) in EG and VG clusters and subsets in ST1 MW2 3-R vs. 3-S.

Under-expressed mRNAs (A) and dysregulated asRNAs (B) in EG and VG clusters and subsets in ST1 MW2 3-R vs. 3-S were shown in Figure 3.

### 2.3. Transcriptional Trend Validation

The transcriptional trend validation of murF, mprF, and dltA coding mRNAs confirmed the differential expression trends of the overall average of mRNA and asRNAs, relative to a single gene, recovered in RNA-seq outputs in each strain-pair, however, partially previously described [23]. On the contrary, the transcriptional trend validation of sucA asRNAs confirmed the differential expression trend of an exclusive asRNA detected in RNA-seq experiments in each strain-pair, partially previously reported [23].

All real-time qPCR data were reported as comparisons of the R vs. S strain in each strain pair; transcriptomic Log2 fold-changes vs. real-time qPCR are detailed in Table S3.

## 3. Discussion

The balance between AMR and fitness/virulence in MRSA is highly complex [24]. Various factors, including genomic backgrounds, responses to antimicrobial exposure, mechanisms of AMR resistance, and the nature of infections (acute or chronic), can influence this interplay. AMR often comes at the cost of overall bacterial fitness, affecting growth ability, competitiveness, and virulence, influencing the microorganism’s transmission and spread in drug-free environments [13,25].

The FC molecular basis remains poorly characterized and understood. AMR can reduce biological fitness as antimicrobials target essential bacterial functions such as cell wall biosynthesis, chromosome supercoiling, RNA transcription, and protein synthesis. The core-genome and, thus, its essential genes, control these critical pathways necessary for survival and growth. This means that mutations or changes in their expression can significantly impact fitness [6]. Similarly, virulence is closely linked to pathogen fitness [24], and, hence, can be subject to selection. For directly transmitted pathogens, higher growth-rates within the host are linked to higher transmission-rates and better microorganism fitness [26]. 

Based on these observations, for the first time, our study tried to close the knowledge gap regarding the interconnections among XDR MOA-related AMR-mechanisms, genomic proneness, the FC omic basis, and compensatory mutational and transcriptional adaptations to acquire and trade off XDR in high-risk HA-, LA-, and CA-MRSA.

This research investigated three isogenic high-risk HA-MRSA, LA-MRSA, and CA-MRSA clones with different genomic backgrounds, expressing DAP/DAL-GLY resistance, paying increasing AMR FCs to explore the FC omic molecular basis, MOA-related AMR-mechanisms, the “FC-compensatory” adaptations, and genomic proneness in this extreme drug resistance (XDR).

Our integrated omic data underpinned the molecular role of the EG and VG gene pools as strategic ‘hot spots’ of compensatory mutational and transcriptional adaptations in the transition from susceptibility to mono- or cross-resistance to second-line DAP/DAL and first-line GLY antimicrobials in high-risk HA-MRSA, LA-MRSA and CA-MRSA acquiring a low or high FC burden.

Firstly, it was mandatory to characterize the MOA-related resistance mechanisms, responsible for the mono-DAP-R in ST-5 N315 HA-GSSA, cross-DAP-R/hGISA in ST398 LA-MRSA, and cross-DAP-R/DAL-R/GISA in ST-1 MW2 CA-MRSA, to outline their impact on vital cellular functions and their association with the omic FC burden in a specific genomic background.

In ST-5 N315 HA-MRSA, mono-DAP-R appeared to be associated with a dual drug-repulsion resistance-mechanism based on “target-enrichment” and “positive-charge enrichment” features. This was related to a concomitant over-expression of tarL/F (target-enrichment) and dltA/C (positive-charge enrichment), implicated in WTA biosynthesis and modification, coupled to an over-expression of the phospholipid biosynthesis-related gene pgsA (target-enrichment); however, these were not co-expressed with mprF (positive-charge enrichment). These changes can putatively result in increases to the WTA abundance and their D-alanylation rate, as well as an exclusive putative increase in the membrane-bound phosphatidylglycerol (PG) amount without an increase in its L-lysinylation rate. This differential expression profile supports a putative overall increase in cell-wall and cell-membrane positive net-charge, even though by different mechanisms. Despite our analysis identifying an MI-nsSNP T345I in MprF, its role remained unclear. This mutation is predicted to decrease the protein stability and functionality (−0.86 ΔΔG), supporting a possible decline in the L-lysinylation rate of membrane-bound PG. This could reduce the positive net-charge of the cell-envelope. These findings aligned with previous studies that question the direct functional impact of MprF SNPs on resistance mechanisms [27].

The acquisition of mono-DAP-R in the ST-5 N315 HA-MRSA genomic background resulted in very low FCs, with minimal reductions in growth ability, competitiveness, and virulence (both in vitro and in vivo models) [13]. This correlated with the reported extra-small-scale mutation- and transcription-driven FC burden. Mutation-driven FC burden was only in charge of two MI-nsSNPs in genes regulating the cell-envelope charge (*mpr*F) and peptidoglycan metabolism (*fmh*B). The transcription-driven FC burden was related to an under-expression of an extra-small pool of EG and VG mRNAs and asRNAs. mRNA under-expression was observed, in decreasing order, in the protein synthesis - (mainly in the ribosomal protein EG subset), unknown/other, followed by the cell-envelope (mainly in the peptidoglycan along with cell division EG subsets), and carbon metabolism in the (mainly in glycolysis EG subset) EG clusters. Interestingly, the lowest under-expression trend was found in the VG cluster. Consequently, in ST-5 N315 HA-MRSA, it can be speculated that there was a minor amount of different coding mRNAs, including the ribosomal protein coding targets, i.e., RpsS (small ribosomal subunit protein uS19), involved in a complex with S13 that binds strongly to the 16S ribosomal RNA, RplS (large ribosomal subunit protein bL19), involved in the structure and function of the aminoacyl-tRNA binding site, and RplD (large ribosomal subunit protein uL4) and RplC (large ribosomal subunit protein uL3), implicated in the assembly of the ribosomal 50S subunit; the Unknown targets, i.e., ppaC, plsY, pmtD, ybeY, SA0003, SA0878, SA1445; the peptidoglycan biosynthesis targets, i.e., MurT (Lipid-II isoglutaminyl-synthase complex) catalyzing the formation of alpha-D-isoglutamine in the cell wall lipid II stem peptide and femX (Lipid II–glycine glycyltransferase), responsible for the incorporation of the first glycine of the pentaglycine interpeptide bridge; the cell-division-related genes, i.e., FtsZ (an essential protein that forms a contractile ring structure (Z ring) at the cell division site) and gidA (NAD-binding protein), which forms tRNA-cmnm5s2U34; the glycolysis-related targets, i.e., Prs (ribose-phosphate pyrophosphokinase), involved in the biosynthesis of the central metabolite phospho-alpha-D-ribosyl-1-pyrophosphate (PRPP); and Eno (Enolase), catalyzing the reversible conversion of 2-phosphoglycerate into phosphoenolpyruvate.

Concurrently, dysregulations via asRNAs were found to be prevalent in the following clusters and subsets: in the protein synthesis EG cluster, mainly in the tRNA synthesis subset, via the under-expression of ileS (isoleucine–tRNA ligase), gatC (aspartyl/glutamyl-tRNA(Asn/Gln) amidotransferase subunit C), and the over-expression of lysS (Lysine–tRNA ligase), trpS (tryptophan–tRNA ligase), alaS (alanine–tRNA ligase), glyS (Glycine–tRNA ligase), and in the ribosomal proteins, via the over-expression of rpsF, rpsR, rplA, rpmB, rplY; in the cell-envelope metabolism EG cluster, mainly in peptidoglycan biosynthesis, via the over-expression of pbp2 (peptidoglycan carboxypeptidase), murT (Lipid II isoglutaminyl synthase), murB (UDP-N-acetylenolpyruvoylglucosamine reductase), and ddl (D-alanine–D-alanine ligase); as well as in the teichoic acid modification subset, via the over-expression of tagH (teichoic acids export ATP-binding protein), llm (lipophilic protein), dltA (D-alanine–D-alanyl carrier protein ligase), and cell-wall amino sugar subsets via the under-expression of glmS (glutamine–fructose-6-phosphate) and the over-expression of glmS, glmU (bifunctional protein GlmU), catalyzing the last two sequential reactions in the de novo biosynthetic pathway for the UDP-N-acetylglucosamine (UDP-GlcNAc); in the unknown/other EG cluster; in the RNA-metabolism EG cluster, mainly in the RNA-regulation subset, via the under-expression of greA, spxA and over-expression of fur (ferric uptake regulation protein), lexA (LexA repressor), repressing the genes involved in the response to DNA damage (SOS response), as well as in basic transcription machinery, via the over-expression of rpoB (DNA-directed RNA polymerase subunit beta) and rpoC (DNA-directed RNA polymerase subunit beta’); and in the virulome cluster, mainly in the adhesion subset, via the under-expression and over-expression of two different loci in sdrC, and the over-expression of clfB, clfA, and icaA–icaD.

These transcriptomic data demonstrated the smallest decrease in the transcriptional rate of the EG and VG clusters associated with the mono-DAP-R acquisition in ST-5 N315 HA-MRSA.

This relatively minimal mutation- and transcription-driven omic FC burden provided significant molecular evidence supporting and agreeing with the very low in vivo FC burden (very low reduction in growth performance, competitiveness, and virulence) as previously outlined in ST5 N315 DAP-R HA-MRSA [13].

Regarding ST398 DAP-R LA-hGISA, cross-DAP-R/hGISA could seem putatively associated with a GLY-trapping within a thicker cell wall and an alternative “target-enrichment” related to a DAP charge-repulsion mechanism. hGISA was caused by increased peptidoglycan monomers, leading to a thickened cell wall, determining a putative GLY entrapment. This occurred via the over-expression of genes involved in the cell-envelope metabolism, particularly those associated with peptidoglycan biosynthesis, such as murI (glutamate racemase) and murA (UDP-N-acetylglucosamine 1-carboxyvinyltransferase 1), which plays a central role in the core steps of biosynthesis.

Similarly, DAP-R seems determined by a “target-enrichment” mechanism leading to a more copious amount of WTAs, despite not being associated with their increased D-alanylation rate. Similarly, an increased positive cell wall net-charge seems determined by an increased target abundance. This occurred through the over-expression of WTA modification and biosynthesis EG tarL (CDP-glycerol glycerophosphotransferase family protein), not co-expressed with the dlt-operon. 

On the contrary, no charge repulsion mechanism due to mprF differential expression was demonstrated, and the role of MprF S295L remains dubious, as commented upon in previous publications [27].

The acquisition of cross-DAP-R/hGISA in ST398 LA-MRSA conferred low in vivo FCs, with a medium reduction in growth ability, a low decrease in competitiveness, and no effects on virulence (both in vitro and in vivo model) [13]. This corresponded to a small mutation- and transcription-driven FC burden, marked by the under-expression of a medium-sized set of EG and VG targets. In detail, the mutation-driven FC burden was linked to four EG MI-SNPs, including a gene regulating the cell-envelope charge and an unknown EG exported protein (SAPIG1881). Interestingly, the two remaining nsSNPs were found as compensatory mutations in the basic transcription machinery-EGs, rpoB (DNA-directed RNA polymerase subunit beta), and rpoC (DNA-directed RNA polymerase subunit beta’). One VG mutation was, uniquely, in the cell-wall-associated fibronectin-binding protein coding ebh. The transcription-driven FC-burden was mainly due to a small-size under-expression of EG and VG mRNAs as well as asRNAs. This was observed, in decreasing order, in the protein synthesis (mainly in the tRNA synthetase, ribosomal protein, and protein-folding EG subsets), unknown, cofactor-metabolism (mainly in FE sulphate and folate EG subsets), and DNA-metabolism EG cluster (mainly in the DNA-replication EG subset). Interestingly, a very-small-scale differential expression was defined in the VG cluster, with only three under-expressed virulence factors in the immune evasion, exoenzyme, and exotoxin VG subsets, in agreement with the lack of FCs in the in vitro and in vivo virulence [13], and the RNA metabolism EG cluster (mainly in the RNA-modification subset). In ST398 DAP-R LA-hGISA, lower coding mRNA can be reported among in the following: in tRNA-synthetases, i.e., serS (serine-tRNA synthetase) and gatC (asparaginyl-tRNA or glutaminyl-tRNA synthetase); in ribosomal proteins, i.e., rpmF (a large ribosomal subunit protein bL32 and rpmA (large ribosomal subunit protein bL27); in protein-folding targets, i.e., GrpE related to the hyperosmotic and heat shock by preventing the aggregation of stress-denatured proteins and GroEL; in unknown, i.e., ppaC, rplGA, SAPIG1610, SAPIG1681; in Fe-sulfate cofactor targets, i.e., SufC (Fe-S cluster assembly ATPase) and IscS (probable tRNA sulfurtransferase) catalyzing the ATP-dependent transfer of a sulfur to tRNA to produce 4-thiouridine in position 8 of tRNAs, which functions as a near-UV photosensor; in the folate-cluster target FolE2 (GTP cyclohydrolase) and folC (tetrahydrofolate synthase); in the DNA-replication targets, i.e., dnaG (DNA primase), an RNA polymerase, catalyzes the synthesis of short RNA molecules used as primers for DNA polymerase during DNA replication, such as holA (DNA polymerase III subunit delta), and DNA packaging and segregation hu (DNA-binding protein HU); in virulome, i.e., immune evasion SAPIG0171 capsular polysaccharide biosynthesis protein, exoenzyme SAPIG2721 GehA lipase, and exotoxin SAPIG1158 alpha-hemolysin, as well as in the RNA metabolism, mainly in the RNA modification rnjA (ribonuclease J 2), rimM (ribosome maturation factor RimM), and trmD (tRNA (guanine-N(1)-)-methyltransferase).

Concurrently, dysregulations via asRNAs were demonstrated in the RNA-metabolism EG cluster, mainly in the RNA-modification subset, via the under-expression of rnjA (Ribonuclease J 2), involved in the maturation of rRNA, and in some organisms, mRNA maturation and/or decay, as well as in the RNA-regulation subset, via the under-expression of fur (ferric uptake regulation protein) and the over-expression of spxA (global transcriptional regulator Spx), which were also demonstrated to be involved in the global transcriptional regulator—which plays a key role in the stress response. Dysregulations via asRNAs were also demonstrated in the following subsets: in the basic transcription machinery subset, via the under-expression of rpoB (DNA-directed RNA polymerase subunit beta), catalyzing the transcription of DNA into RNA using the four ribonucleoside triphosphates as substrates; in the protein synthesis subset, via the dysregulation in ribosomal protein rpsD and the over-expression of the translation factor dtd (D-aminoacyl-tRNA deacylase) and fusA (Elongation factor G); in DNA-metabolism EG clusters, i.e., the DNA-replication subset, via the under-expression of ssb (single-stranded DNA-binding protein), holA (DNA polymerase III subunit delta), and pcrA Elicase (ATP-dependent DNA helicase PcrA); in the virulome cluster, via the under-expression of sdrC, as well as in the over-expression of atl, sdrD, adhesins, and in the immune evasion of capC.

These transcriptomic data evidenced a small-scale reduction in the EG and VG expression profile related to the cross-DAP-R/hGISA in ST398 LA-MRSA.

This low mutation- and transcription-driven omic FC-burden provides coherent molecular evidence supporting and agreeing with the low in vivo FC burden characterizing ST398 DAP-R LA-hGISA [13].

Concerning ST-1 MW2 CA-GISA, the cross-DAP-R/DAL-R/GISA in this genomic background seems to be associated with the transcription-driven MOA-related AMR-mechanisms. DAP-R is related to a dual mechanism of “positive-charge enrichment” by a co-overexpression of dltA/B/D and mprF. This speculatively increased the cell wall and cell-membrane positive net-charge, resulting in a resistance mechanism based on charge-repulsion for a “target-modification enrichment” mechanism. Although our data showed an MI-nsSNP, determining T345A in MprF, its precise role is, similarly, unclear. This mutation resulted in a greater decrease in protein stability (−1.62 DDG change), suggesting a potential reduction in MprF functionality. The putative reduced L-lysinylation rate of membrane-bound PG, caused by the T345A in MprF, could lower the cell’s positive net charge, weakening the charge-repulsion resistance mechanism against positively charged compounds such as GLYs or DAP. However, mprF co-overexpression might compensate for the decreased protein functionality, enabling it to still play a key role in maintaining resistance to DAP. In addition, the low availability of the D-Ala-D-Ala terminus precursor, due to the under-expression of alanine racemase (alr) and D-Ala-D-Ala ligase (ddl), could result in a reduced teichoic acid D-alanylation rate, weakening the charge-repulsion resistance mechanism. Consequently, it can be hypothesized that this deficiency was compensated by the co-overexpression of dltA/B/D and mprF, which may help retain resistance. DAL-R/GISA was previously related to a key MOA-related target differential expression, which includes the under-expression of the alanine racemase (alr) and D-Ala-D-Ala ligase (ddl) involved in peptidoglycan biosynthesis. These genes are essential for the D-Ala-D-Ala terminus synthesis in the peptidoglycan monomer, which plays a critical role in transpeptidation. This expression profile indicates a reduced availability of D-Ala precursors and decreased D-Ala-D-Ala binding, which are targets of DAL and GLY antimicrobials. Interestingly, this was associated with numerous accessory dysregulated pathways supporting the key one, as previously published [23].

In ST-1 MW2 CA-GISA, the acquisition of cross-DAP-R/DAL-R/GISA, resulting in high in vivo FCs (severely impaired growth performance, reduced competitiveness, and decreased in vitro and in vivo virulence) [13], was associated with a large-scale mutation-driven and transcription-driven FC burden by large-scale under-expression of the EG and VG targets.

The mutation-driven FC burden was in charge of MI-nsSNPs in the cell-envelope charge and peptidoglycan biosynthesis EG subsets (similar to DAP-R HA-GSSA) including the T345A in MprF (DAP/GLY-R), I121N in MurG (indirectly supporting GLY-RS), F74L in TagH (teichoic acid biosynthesis), H481Y in RpoB (RIF-R and transcription), and in an unknown/other EG cluster, i.e., I186M in GdpG (β-lactam resistance and GLY-cross-resistance). Furthermore, wild-type-revertant mutations included V329G in GlnA (glutamine biosynthesis, carbon/intermediary metabolism) and N177K in RecU (DNA metabolism/DNA packaging). 

An additional mutation-driven burden was found in the VG cluster. This included Y130H in Cap8H and V120G in Cap8K (capsular polysaccharide synthesis enzymes), T1313S SdrD (serine-aspartate repeat adhesion protein), V1768D in Ebh (cell-wall-associated fibronectin-binding protein), and only one wild-type-revertant MI-nsSNP D635E in the ClfB wild type (adhesion clumping factor B). 

The transcription-driven FC burden was predominantly characterized by massive under-expression across all EG clusters and VG clusters. EG mRNA under-expression, in decreasing order, was most notable in the protein synthesis- (predominantly in the ribosomal protein EG subset), VG (mainly in the exotoxin, immune evasion subsets and equally adhesins), and in unknown/other clusters. A smaller under-expression was observed in the carbon metabolism, DNA-, cofactor metabolism, and cell-envelope EG clusters. In ST-1 MW2 CA-GISA, a lower coding mRNA amount was mainly found for the gene-pool including rpsF, rpsB, rpsS, rplU, rplM, rplF, rpsC, and rplV (ribosomal proteins); metS, lysS, and thrS (t-RNA synthetase); in virulence genes, i.e., hla, hld, hlgB, hlgC (hemolysin), seh (enterotoxins), in sdrC, sdrD, eap/map, atl (Adhesins), and in esaA, cap8P (immune evasion); in unknown targets, i.e., MW1973, MW0014, MW0708, MW1975, MW2090, MW1106, MW1151, MW2218, and MW1548; in glycolysis-related genes, i.e., pgk (phosphoglycerate kinase), tpiA (triosephosphate isomerase), pgi (glucose-6-phosphate isomerase), and gap (glyceraldehyde-3-phosphate dehydrogenase 1); in DNA replication genes, i.e., holA (DNA polymerase III subunit delta), dnaI (primosomal protein), dnaB (replicative DNA helicase), and pcrA (ATP-dependent DNA helicase PcrA); in folate-cofactor targets, i.e., folB (dihydroneopterin aldolase) and folE2 (GTP cyclohydrolase FolE2); peptidoglycan biosynthesis genes, i.e., murI (glutamate racemase) and ddl (D-alanine–D-alanine ligase).

Additionally, putative key transcriptional dysregulations via asRNAs were progressively found in the protein synthesis, mainly in the following subsets: in the ribosomal protein EG subset via the under-expressions in rpmF, rpsD, rplO, rplN, rpmC, rpsS, rpsB, and the over-expression of rpsL; in the t-RNA synthetase subset, via the under-expressions in glyS, alaS, thrS, and the over-expression of valS; in unknown/other, i.e., obgE, engA, MW0544, MW0837, MW1104, MW1350, MW1727, MW1860, MW0681, and OE in MW1860, MW1240, MW0544, MW1009, MW1692, MW2353, and MW1872; in the VG cluster, mainly in the adhesin subset, via the under-expressions in sdrD, clfA, clfB, atl, can, and the over-expression of sdrH; in the exoenzyme subset, via the under-expression of lip, sspA, splB, and the over-expression of splC; in the folate subset, via under the expression of dfrA, and the dysregulation in folC; in the Fe/sulphate subset, via the under-expression of sufS (cysteine desulfurase), sufU (iron–sulfur cluster assembly scaffold protein IscU), and the over-expression of sufS; in the cell-envelope EG cluster, mainly in the peptidoglycan biosynthesis subset, via the under-expression of murF (UDP-N-acetylmuramoyl-L-alanyl-D-glutamate–L-lysine ligase), murT (Lipid II isoglutaminyl synthase), and ddl (D-alanine–D-alanine ligase); in the DNA-metabolism EG cluster, mainly in the DNA-replication subset, via the under-expression of dnaA (chromosomal replication initiator protein DnaA), dnaN (Holliday junction branch migration complex subunit RuvB), and dysregulation in ssb (single-stranded DNA-binding protein); and in the DNA packaging and segregation subset, via the under-expression of gyrA (DNA gyrase subunit A), gyrB (DNA gyrase subunit B), topA (DNA topoisomerase 1), and the over-expression of recU (Holliday junction resolvase RecU). A smaller dysregulation occurred in the RNA-metabolism EG cluster, mainly in the following subsets: in the basic transcription machinery subset, via the under-expression of rpoB (DNA-directed RNA polymerase subunit beta) and the over-expression of two diverse rpoB and rpoC (DNA-directed RNA polymerase subunit beta’); in the nucleotide metabolism EG, mainly in the purine and pyrimidine biosynthesis subset, via the under-expression of gmk (guanylate kinase), nrdE (Ribonucleoside-diphosphate reductase), and the over-expression of nrdI (Protein NrdI) and pyrG (CTP synthase); and in the carbon-metabolism EG cluster, mainly in the glycolysis-subset, via the under-expression of pykA (pyruvate kinase), fbaA (fructose-bisphosphate aldolase), and Eno (Enolase).

Transcriptomics demonstrates that cross-DAP-R/DAL-R/GISA required a drastic decrease in the overall transcription trend of essential and virulence genes, in contrast to the mono-DAP-R and cross-DAP/hGISA acquisition. 

This maximal mutation- and transcription-driven omic FC burden provides robust molecular-level supporting evidence, in agreement with the high in vivo FC-burden (huge/high decrease in growth-performance, competitiveness, and virulence) previously reported in ST-1 MW2 CA-GISA [13].

Noteworthy considerations need to be addressed about the molecular basis of genomic proneness and its “compensatory-skills” to acquire and maintain the omic adaptations necessary for XDR.

First, the FC compensatory adaptations are not random but, on the contrary, they systematically appear in key genomic regions—particularly those involved in protein synthesis, cell-envelope metabolism (a hub for lipopeptide, β-lactam, and glycopeptide resistance), and the virulome—revealing a harmonized evolutionary response to antibiotic pressure.

In the core-genome EG clusters, we observed coordinated transcription-shifts and allelic diversification, suggesting an evolved genomic plasticity that supports adaptability in variable-scale prone genomes, particularly those undergoing genome reduction, rearrangement, or horizontal acquisition events. These compensatory mechanisms are amplified in high-risk clones from hospital-acquired (HA), livestock-associated (LA), and community-acquired (CA) MRSA lineages, each presenting distinct environmental and host-imposed selective landscapes.

In LA-MRSA, HA-MRSA, and CA-GISA, the EG clusters related to protein synthesis, cell envelope, and virulence play a key role in the omic basis of FCs. 

From a molecular point of view, it remains to be considered that the differential expression can lead to a major or minor amount of the mRNA target genes, whilst the differential expression of regulatory asRNAs can lead to the up- or down-regulation of its regulated targets or pathways via an increase or decrease in transcriptional or translational rate. Furthermore, asRNAs can regulate gene expression by several mechanisms. One is a transcriptional interference, i.e., RNA polymerase that transcribes the asRNA physically blocks the transcription of the sense-strand. Post-transcriptionally, asRNAs can alter the structure of the sense RNA, affecting its translation or promoting degradation by RNases. RNase III, which cleaves double-stranded RNA, plays a key role in this process [28,29,30,31,32,33].

Looking only at the mostly involved clusters, the protein synthesis of the EG cluster involves pathways such as ribosomal proteins, t-RNA synthetase, translocation factors, protein folding, and protein translocation. Adaptations in these pathways can disrupt normal protein synthesis, reducing translation efficiency and causing defective protein production, misfolded proteins, and impairing translation. These changes may require additional energy, negatively affecting fitness.

The cell-envelope metabolism EG cluster, enriched with seven essential pathways such as peptidoglycan biosynthesis and cell-envelope modification, can confer mono or cross DAP and DAL/GLY-R resistance. These adaptations alter cell morphology, impair envelope integrity, reduce stress resistance, and compromise growth, all linked to adaptive lipoglycopeptide (DAL)/glycopeptide (GLY) resistance mechanisms.

The VG cluster involves adaptations in exotoxin, immune evasion enzymes, adhesins, exoenzymes, biofilm, superantigens, toxins, and secretion systems, which can drastically change virulence and pathogenicity.

Successively, it is mandatory to point out the key role of the occurrence of the mitigating molecular factors, i.e., compensatory mutations or “transcription-shifts”. In ST-1 MW2 CA-GISA, the high-impact nsSNP in agrA (Arg170*), quorum sensing master regulator of the staphylococcal virulence, the MI-nsSNP in RNA polymerase beta subunit coding gene, rpoB, together with the large-size transcription-shift confer to this genome high-proneness and compensatory skills to balance FCs due to XDR. ST-1 MW2 CA-MRSA results in a highly prone genome able to support and balance the massive XDR omic burden, despite its intrinsic nature to carry a lower AMR rate, sufficient for spreading in low antimicrobial pressure community settings. Based on actual knowledge, the agrA and rpoB compensatory mutations drastically reduced virulence and transcription rate, representing compensatory features allowing the chance to use cell resources to do other things, i.e., shifting the metabolism towards the new XDR-related pathways [12]. The acquisition of cross-DAP-R/DAL-R/GISA required complex and massive mutational and transcriptional rearrangements. These support the XDR mechanism, significantly reducing microorganisms’ fitness in free-drug conditions. The adaptations leading to FCs by high growth retardation, poor competitiveness, and low virulence impose an energetic and metabolic burden, compromising vital functions and virulence. To express cross-DAP/DAL/GLY MOA-related AMR mechanisms, CA-GISA requires additional efforts to synthesize the new enzymes necessary to activate the complex network of metabolic resistance pathways and to reallocate the available resources. This represents a critical factor in microbial competition [34]. At the phenotypic level, this matches the observed slowing of growth and poor competitiveness [13], whilst at the omic level, it can align with transcriptional compensatory adaptations in essential genes involved in vital pathways, such as protein synthesis, cell-envelope metabolism, cofactor biosynthesis, DNA–RNA processes, carbon and nucleotide metabolism, and the respiratory pathway, all influencing growth performance.

In the ST398 mono-DAP-R LA-MRSA genomic background, the MI-nsSNPs in genes coding the beta and beta’ subunits of the RNA polymerase, rpoB and rpoC, as well as the small-size transcription-shifts confers to this genome a “FC-compensatory” moderate-proneness and, thus, compensatory skills to balance the costs for the XDR acquisition. The ST398 LA-MRSA genomic background indeed acquires a lower resistance-rate, i.e., a cross-DAP-R/hGISA, requiring a smaller-scale mutation- and transcription-driven burden. The cross-DAP-R/hGISA mechanism is due to a different resistance mechanism caused by GLY-trapping and a “target-enrichment” DAP-alternative repulsion mechanism. The former arises from the differential expression of two genes; however, whilst this affects a high-interconnected pathway such as the peptidoglycan biosynthesis, contrarily, the latter results from a single-gene differential expression (tarL), implicated in a more tailored pathway such as the WTA modification and biosynthesis.

In the ST-5 N315 HA-MRSA genomic background, no compensatory mutations were observed; thus, the extra-small-size compensatory transcriptional shifts allow the genome a low proneness to balance the costs for XDR. The ST-5 N315 HA-MRSA genomic background can face the acquisition of mono-DAP-R, requiring an extra-small-scale and transcriptional burden. The DAP-R mechanism is, in fact, due to the over-expression of tarF/L and dltA/C, both exclusively tailored to the cell-envelope’s positive net-charge modifications.

Trying to close the gap between omic FC and their clinical and molecular implications, our findings underscore the significance of the functional compensatory landscape (“FC-compensatory” mechanisms) within essential genomic modules—specifically those involved in protein synthesis, cell-envelope metabolism, and the virulome—in shaping the evolution of extreme drug resistance (XDR) in MRSA. The existence of FC compensatory adaptations across these genomic compartments facilitates MRSA in achieving a balance between resistance and fitness, thereby aiming to optimize persistence, virulence, and transmissibility.

These compensatory landscapes appear to be a critical evolutionary response to the selective pressures exerted by antimicrobials targeting the multi-faceted mechanisms of action (MOAs) of frontline antibiotics. This supports a model in which “FC compensatory” features are not merely casual, but integral to the success of XDR MRSA in diverse ecological contexts. Adapting critical cellular functions impacted by resistance mechanisms, these compensatory elements aim to optimize the persistence and transmissibility of high-risk MRSA lineages, thereby posing an ongoing threat to patient outcomes, and thus, to human health.

In the clinical context, there is an urgent need for therapeutic strategies that anticipate or disrupt such compensatory adaptations, potentially targeting these molecular trade-offs to reinstate susceptibility and to limit the spread of XDR MRSA. This significantly undermines treatment efficacy, increases the burden of care, and leads to poorer outcomes for patients across all exposure contexts—hospital, livestock-associated, and community-acquired.

A comprehensive understanding of the compensatory landscape has the potential to enhance risk stratification, guide therapeutic choices, and most significantly, inform the development of treatments that target these adaptations. This could affect the pathogen's ability to sustain resistance without sacrificing virulence or metabolic integrity. Furthermore, such treatments could reverse the rising tide of treatment failure and improve patient outcomes in the face of an evolving and deeply adaptive pathogen. The integration of compensatory dynamics into our understanding of resistance evolution offers a refined framework for predicting MRSA behavior across different epidemiological niches. Furthermore, it provides a foundation for the rational design of next-generation antimicrobials and diagnostics aimed at intercepting adaptive resilience in pathogenic bacteria.

In conclusion, our data first demonstrated the role of compensatory features appearing in protein synthesis, cell-envelope metabolism, EG clusters, and virulome as key mediators of “FC compensatory” adaptations related to the XDR MOA-related resistance in variable-scale prone genomes of high-risk HA-, LA-, and CA-MRSA.

## 4. Materials and Methods

### 4.1. Bacterial Strains

Three clinical S. aureus isogenic strain pairs (1-S and 1-R, 2-S and 2-R, and 3-S and 3-R), models of the most widespread and high-risk MRSA clones, isolated under DAP or GLY mono or combined therapy and acquiring stepwise cross-DAL/GLY and DAP-R, were included in the study. Second/first-line AMR, genomic characterization, and FC burden were previously published and shown in Table 1 and Table 3 [13].

### 4.2. Whole-Genome Sequencing 

Following the manufacturer’s instructions, genomic DNA was extracted using the PureLink Genomic DNA Mini Kit (Invitrogen, Waltham, MA, USA), as previously published [12,13,23].

### 4.3. Whole-Genome Sequencing (WGS) 

Whole-genome sequencing (WGS) was performed with an Illumina MiSeq system using paired-end (PE) read libraries prepared with the Nextera XT DNA Library Prep Kit (Illumina, San Diego, CA, USA). The libraries were evaluated using published methods [35]. The raw reads were assessed using FastQC (v.0.11.7). The cutadapter tool (v.1.16), implemented in Python (v.3.5.2), was used to remove residual PCR primers and to filter low-quality bases (Q_score < 30) and short reads (<150 bp) [35]. The filtered trimmed reads were included in the downstream analysis.

### 4.4. De Novo Genome Assembly

Using the SPAdes program (v3.12.0), a de novo genome assembly was carried out. Each sample was given a contigs file by the SPAdes program, and post-assembly controls and metrics were assessed using Quast software (v4.6.3), as previously published [12,13,23,36]. 

### 4.5. Gene Annotation

The Prokka software (1.14.6) was used to analyze the assembled contigs to predict genes and to annotate using a core set of conserved prokaryotic genes, as previously published [12,13,23,36].

### 4.6. Essential Gene Clusterization

Essential gene clusterization was performed according to the grouping of essential gene categorization proposed by Chaudhuri R.R. et al. [37].

### 4.7. Essential Gene Single-Nucleotide Polymorphisms (SNPs)

On N315 (BA000018.3) RefGen in 1-S/R strain pair, ST398 (AM990992.1) RefGen in 2-S/R, MW2 RefGen (BA000033.2) in 3-S/R, snpEff (v4.3) was used to predict the core genome single-nucleotide polymorphisms [12,13,23,36]. 

Variant calling versus N315 (BA000018.3) RefGen in 1-S/R strain pair, ST398 (AM990992.1) RefGen in 2-S/R, MW2 RefGen (BA000033.2), and in 3-S/R was performed to predict the whole-genome nsSNPs, as previously published [12,23]. Sanger sequencing was used to confirm the non-synonymous SNPs.

### 4.8. Essential Gene and Virulence Single-Nucleotide Polymorphism Effect Prediction Criteria

SnpEff (v. 4.3T) assessed the essential gene SNP impact prediction. According to previously published criteria, high-impact (HI) variants are thought to be disruptive, presumably leading to protein truncation, loss of function, or inducing non-sense-mediated decay; low-impact (LI) variants are considered to be generally benign or unlikely to alter protein behavior; moderate-impact (MI) variants are a non-disruptive variation with a potential to alter protein performance; the modifier effect (MOD-I), usually non-coding variations or variants impacting non-coding genes, whose impact projections are uncertain because there are no supporting data [12,36,38]. Only EG and the virulence gene (VG) cluster nsSNPs with high (HI) and moderate (MI) effects were considered and subsequently reported.

### 4.9. I-Mutant Neural Network-Based Predictor of Protein Stability Changes upon Mutation

I-Mutant Suite Predictor of the effects of single-point protein mutation online software was used to predict the impact on the protein function (https://mupro.proteomics.ics.uci.edu) [39].

### 4.10. RNA-Seq

#### 4.10.1. RNA-Seq Cultures

A 5 × 10^5^ CFU/mL inoculum was prepared by diluting an aliquot of an overnight culture 1:50 in 30 mL of brain heart infusion (BHI) in a sterile 50 mL flask (OD600 nm 0.05). Cells were cultivated until the exponential growth phase (OD_600_ 0.5, 2 × 10^8^ CFU/mL for 3–4 h) with shaking (250 rpm) and typical atmospheric conditions at 37 °C. Colony counting was used to calculate the cell density after plating onto Mueller–Hinton (MH) agar and incubation. To preserve RNA integrity, RNA extraction started right away after cell collection [12,23,36].

#### 4.10.2. RNA Extraction

To maximize the gathered RNA-seq data, tailored RNA extractions for the Tru-Seq Library and Short-Insert Library were carried out with the specified methods, as previously described [12,23,36].

#### 4.10.3. RNA-Seq Libraries

On the Illumina Mi-seq sequencing platform, RNA-seq was performed. A single-end library with 50 bp reads (SI, Short-Insert Library) and a paired-end read library with 150 bp reads (TS, Tru-Seq Library) with an average insert size of 350–400 bp were used in two repetitions to improve RNA-seq data [12,23,36].

#### 4.10.4. Tru-Seq and Short-Insert Library Raw Read Post-Processing 

After the sequence data generation, raw readings were processed using FastQC (v.0.11.2) to evaluate the data quality and Trimmomatic (v.0.33.2) to trim the sequenced reads, removing just the sequencing adapters for PE reads. Phred’s minimum base quality requirement of 15 over a four-base sliding window has to be met. The downstream analysis only included sequences longer than 36 nucleotides, and, similarly, only trimmed reads were used in the downstream analysis [12,23,36]. Similarly, single-end reads were trimmed using Trimmomatic (v.0.33.2), which required a minimum base quality of 15 (on the Phred scale) and a minimum read length of 15 nucleotides. In the downstream analysis, only trimmed reads were used [12,23,36].

#### 4.10.5. Tru-Seq and Short-Insert Read Analysis

TS and SI RNA-seq reads were annotated on S. aureus N315 (BA000018.3), ST398 (AM990992.1), and MW2 (BA000033.2) used as RefGens, as well as transcripts assembled and quantified using Rockhopper (v.2.03) by default parameters [12,23,36]. A statistically significant differential expression was considered for a normalized *p*-value indicated as q-value < 0.01.

#### 4.10.6. Real-Time qPCR Validation

To validate the RNA-seq data, the real-time qPCRs for a set of characteristic transcripts, namely murF, dltA, and mprF, were carried out in the same RNA-seq growth-phase [12,23,36]. For sRNA validation, MW1303 asRNA (sucA), an essential metabolic gene, was used as previously described [12,40]. All sucA asRNA RefGen positions were reported in Table S3. The Log2 fold change was adjusted with the pseudocount parameter for genes with no RPKM or Ct value.

### 4.11. Acronyms

All acronyms were listed in Table S4.

## Figures and Tables

**Table 1:** In vivo FC burden (growth performance, competitiveness, in vitro and in vivo virulence model) in ST5 N315 DAP-R GSSA, ST398 DAP-R hGISA), and ST1 MW2 DAP-R DAL-R GISA.

Strain-Pairs	GRW in S	GRW Burden	GRW in R	COMP in S	COMP Burden	COMP in R	VIR in S	VIR Burden	VIR in R	Total FCs in R
1S/R (ST5 N315 DAP-R GSSA)	Huge	Low	High	Very Low	Very Low	Huge	High	Low	Medium	Very Low
2S/R (ST398 DAP-R hGISA)	High	Medium	Medium	Low	Low	High	Low	Null	Low	Low
3S/R (ST1 MW2 DAP/DAL-R GISA)	Medium	High	Very Low	High	High	Low	High	Huge	Very Low	High

Legend: FC burden was evaluated by changes in growth performance during independent growth (GRW), inter-bacterial competitiveness (COMP) in mixed growth, and virulence (VIR) through virulome size, as well as in vitro and in vivo virulence, comparing resistant (R) and susceptible (S) strains. A growth score burden was assigned according to the rate of growth performance changes, classified as high, medium, or low. A competitiveness score was assigned based on the rate of competitiveness changes, with the following scale: huge, high, medium, low, and very low. A Virulence score was assigned based on the virulome size and the sum of the rates of in-vitro and in-vivo virulence changes, with the following scale: huge, high, medium, low, very low, and null [13].

**Table 2:** Resistance mechanisms related to DAP-R and DAL/GLY-R MOA targets.

	Mutation-Driven Resistance	Transcription-Driven Resistance
Strain Pairs	R vs. S AA Change	R vs. S DRUG-MOA DEGs
ST5 N315 DAP-R DAL-S HA-GSSA (1-R) vs. ST5 N315 DAP-S DAL-S HA-GSSA (1-S)	Daptomycin T345I MprF (−0.86 ΔΔG)	Daptomycin -↑dltA/C, ↑tarF/L, ↑pgsA (Cell-envelope Charge Modification) (Target enrichment and Positive-charge enrichment)
ST398 DAP-R DAL-S LA-hGISA (2-R) vs. ST398 DAP-S DAL-S LA-GSSA (2-S)	Daptomycin S295L MprF (−0.52 ΔΔG)	Glycopeptide-Trapping -↑murA/I (Peptidoglycan Biosynthesis) Daptomycin -↑tarL (Cell-envelope Charge Modification) (Target enrichment)
ST1 MW2 DAP-R DAL-R GISA (3-R) vs. ST1 MW2 DAP-S DAL-S GSSA (3-S)	Daptomycin T345A MprF (−1.62 ΔΔG)	Glycopeptide -↓alr (alanine racemase) -↓ddl -↑murF (Target-Deficit) Daptomycin Positive-charge enrichment -↑mprF, ↑dltA, (Cell-envelope Charge Modification) (Positive-charge enrichment)

**Table 3:** Second/first-line AMR and genomic characterization.

Isogenic Strain Pair	AMR					Second/ First-Line AMR	Genomic Typing					
	VAN MIC mg/L	TEC MIC mg/L	DAL MIC mg/L	DAP MIC mg/L	GLY-PAP	Phenotypes	RefGen	MLST	Spa- Type	Scc-Mec	Agr-Group	*agr*A nsSNP
1-S	1	2	0.047	0.5	GSSA	DAP-S DAL-S GSSA	N315 HA-MRSA	ST-5	t2	II	II	-
1-R	2	2	0.047	2	GSSA	DAP-S DAL-S GSSA						
2-S	1	<0.25	0.023	<0.25	GSSA	DAP-S DAL-S GSSA	ST398 LA-MRSA	ST398	t1939	IVa	I	-
2-R	2	2	0.064	4	hGISA	DAP-R DAL-S hGISA						
3-S	1	1	0.064	0.5	GSSA	DAP-S DAL-S GSSA	MW2 CA -MRSA	ST-1	t127	IVa	III	Arg170 *
3-R	8	32	2	2	GISA	DAP-R DAL-R GISA						

Legend: VAN: vancomycin; TEC: teicoplanin; DAL: dalbavancin; DAP: daptomycin; GLY: glycopeptides; HA-MRSA: hospital acquired methicillin-resistant S. aureus; CA-MRSA: community-associated methicillin-resistant S. aureus; RefGen: reference genome; nsSNPs: nonsynonymous SNPs. * = stop codon.

## Data Availability

The genomic and transcriptomic reads were deposited in the National Centre for Biotechnology Information (NCBI) as follows: 1-S/R BioProjects: PRJNA1216545 (genomes) and PRJNA1241536 (transcriptomes); 2-S/R BioProject: PRJNA49851 (genomes and transcriptomes); 3-S/R BioProject: PRJNA860577 (genomes and transcriptomes).

## Supplementary Materials

The following supporting information can be downloaded at https://www.mdpi.com/article/10.3390/antibiotics14050465/s1, Table S1: nsSNPs in EG- and VG-clusters of MRSA. Table S2: Comparative transcriptomics of 1-S/R, 2-S/R, and 3-S/R strain pairs. Table S3: Genome position and Log2 Fold-Change in transcriptomics and real-time qPCR in all sample. Table S4: List of acronyms in the text.
