# Supplementary material for: Mutation- and Transcription-Driven Omic Burden of Daptomycin/Dalbavancin-R and Glycopeptide-RS Fitness Costs in High-Risk MRSA: A Nexus in Antimicrobial Resistance Mechanisms—Genome Proneness—Compensatory Adaptations
Source: Antibiotics (Basel). 2025 May 2;14(5):465. doi: 10.3390/antibiotics14050465 (PMC12108176; doi:10.3390/antibiotics14050465)
Supplement: Supplementary file 1 [file antibiotics-14-00465-s001.zip › Table S1.pdf]

Table S1. nsSNPs in EG- and VG-clusters of MRSA

1A EG and VG AA Change for MI-nsSNPs in 1-R DAP-R GSSA HA-MRSA vs 1-S DAP-S GSSA HA-MRSA

EG CATEGORY	Gene	AA CHANGE	Product	AMR
**CELL ENVELOPE**				
Cell envelope Charge	*mpr*F	Thr345Ile	Phosphatidylglycerol lysyl-transferase MprF	DAP-R

Legend: EG: essential gene; DAP: daptomycin

1B EG and VG AA Change for revertant MI-nsSNPs to susceptible wild-type in 1-R DAP-R GSSA HA-MRSA vs 1-S DAP-S GSSA HA-MRSA

EG CATEGORY	Gene	AA CHANGE	Product
**CELL ENVELOPE**			
Peptidoglycan biosynthesis	*fmh*B	Lys68Thr	femX/ lipid II:glycine glycyltransferase

Legend: EG: essential gene; DAP: daptomycin

2A EG and VG AA Change for MI-nsSNPs in 2-R hGISA DAP-R LA-MRSA vs 2-S DAP-S GSSA LA-MRSA

EG CATEGORY	Gene	AA CHANGE	Product	AMR
**Cell envelope**				
Cell envelope charge	*mpr*F	Ser295Leu	Phosphatidylglycerol lysyl-transferase MprF	DAP-R
**RNAmetabolism**				
Basic Transcription Machinery	*rpo*C	Val496Ala	DNA directed RNA polymerase beta prime chain, putative *rpo*C	-
**Unknown/other**				
Unknown/Other	SAPIG1881	Tyr66Asn	Probable exported protein	-
**VG CATEGORY**				
Adherence	*ebh*	Ser6725Ile	Cell wall associated fibronectin binding protein *ebh*	

Legend: EG: essential gene; VG: virulence gene; DAP: daptomycin

2B EG and VG AA Change for revertant MI-nsSNPs to susceptible wild-type in 2-R hGISA DAP-R LA-MRSA vs 2-S GSSA DAP-S LA-MRSA

EG CATEGORY	Gene	AA CHANGE	Product	AMR
**RNA metabolism**				
Basic Transcription Machinery	*rpo*B	Asn471Ala	DNA-directed RNA polymerase subunit beta RpoB	RIF-R

Legend: EG: essential gene; RIF: rifampicin

3A EG and VG AA Change for HI-or MI-nsSNPs in 3-R GISA DAP-R CA-MRSA vs 3-S GSSA DAP-S CA-MRSA

EG CATEGORY	Gene	AA CHANGE	Product	AMR
**Cell envelope**				
Cell envelope charge	*mpr*F	Thr345Ala	Phosphatidylglycerol lysyl-transferase MprF	DAP-R
Peptidoglycan biosynthesis	*mur*G	Ile121ASn	Peptidoglycan Lipid II synthesis MurG	GLY-R
**RNA metabolism**				
Basic Transcription Machinery	*rpo*B	His481Tyr	DNA-directed RNA polymerase subunit beta RpoB	RIF-R
**UNKNOWN/OTHER**				
Unknown	*gdp*P	Ile186Met	Cyclic-di-AMP phosphodiesterase GdpP	β-lactam-R
**VG CATEGORY**				
Immune evasion	*cap8*H	Tyr130His	capsular polysaccharide synthesis enzyme Cap8H	-
Immune evasion	*cap8*K	Val120Gly	capsular polysaccharide synthesis enzyme Cap8K	-
Adherence	*sdr*D	Thr1313Se	serine-aspartate repeat proteins SdrD	-
Adherence	*ebh*	Val1768Asp	Cell wall associated fibronectin binding protein ebh	-

Legend: EG: essential gene; VG: virulence gene; DAP: daptomycin; GLY: glycopeptide; RIF: rifampicin

3B EG AA Change for revertant MI-nsSNPs to susceptible wild-type in 3-R GISA DAP-R CA-MRSA vs 3-S GSSA DAP-S CA-MRSA

EG CATEGORY	Gene	AA CHANGE	Product
Cell Envelope/Teichoic acid biosynthesis	*tag*H	Phe74Leu	Teichoic acids export ATP-binding protein TagH
Carbon Metabolism/Intermediary Metabolism	*gln*A	Val329Gly	D-Glutamine synthetase GlnA
DNA metabolism/DNA packaging and segregation	*rec*U	Asn177Lys	Holliday junction resolvase RecU
**VG CATEGORY**			
Adherence	*clf*B	Asp635Glu	Clumping factor B

Legend: EG: essential gene; VG: virulence gene
