# Supplementary material for: Mutation- and Transcription-Driven Omic Burden of Daptomycin/Dalbavancin-R and Glycopeptide-RS Fitness Costs in High-Risk MRSA: A Nexus in Antimicrobial Resistance Mechanisms—Genome Proneness—Compensatory Adaptations
Source: Antibiotics (Basel). 2025 May 2;14(5):465. doi: 10.3390/antibiotics14050465 (PMC12108176; doi:10.3390/antibiotics14050465)
Supplement: Supplementary file 1 [file antibiotics-14-00465-s001.zip › Table S2 .pdf]

Table S2. Comparative transcriptomics of 1-S/R, 2-S/R, and 3-S/R strain pairs

1-R GSSA DAP-R vs 1-S GSSA DAP-S coding up

Category	RefGenN315	Symbol	Product	RPKM 1-S	RPKM 1-R	q-value
**DNA metabolism**						
DNA replication	SA0353	*ssb*	ssDNA-binding protein	0	161	<0.01
DNA replication	SA1391	*dna*G	DNA primase	0	24	<0.01
DNA replication	SA1415	*hol*A	DNA polymerase III subunit delta	0	209	<0.01
DNA replication	SA1720	*lig*A	DNA ligase	0	21	<0.01
**RNA metabolism**						
Basic transcription machinery	SA0501	*rpo*C	DNA-directed RNA polymerase subunit beta'	0	32	<0.01
Basic transcription machinery	SA1390	*sig*A	RNA polymerase sigma factor RpoD	0	74	<0.01
Basic transcription machinery	SA2023	*rpo*A	DNA-directed RNA polymerase subunit alpha	0	53	<0.01
RNA regulation	SA0856	*spx*A	transcriptional regulator Spx	0	226	<0.01
RNA regulation	SA1195	*msr*R	peptide methionine sulfoxide reductase	0	44	<0.01
**Protein synthesis**						
Ribosomal proteins	SA0354	*rps*R	30S ribosomal protein S18	0	172	<0.01
Ribosomal proteins	SA0497	*rpl*J	50S ribosomal protein L10	0	89	<0.01
Ribosomal proteins	SAS052	*rps*D	30S ribosomal protein S4	0	69	<0.01
Ribosomal proteins	SA2025	*rps*M	30S ribosomal protein S13	0	91	<0.01
Ribosomal proteins	SA2031	*rps*E	30S ribosomal protein S5	0	425	<0.01
Ribosomal proteins	SA2037	*rpl*N	50S ribosomal protein L14	0	116	<0.01
Ribosomal proteins	SA2044	*rpl*B	50S ribosomal protein L2	0	97	<0.01
Ribosomal proteins	SA0459	*rpl*Y	50S ribosomal protein L25	0	125	<0.01
Ribosomal proteins	SA0498	*rpl*L	50S ribosomal protein L7/L12	3	12	<0.01
tRNA synthetase	SA0564	*arg*S	arginyl-tRNA synthetase	0	25	<0.01
tRNA synthetase	SA0986	*phe*T	phenylalanyl-tRNA synthetase subunit beta	0	17	<0.01
tRNA synthetase	SA1579	*leu*S	leucyl-tRNA synthetase	0	17	<0.01
tRNA synthetase	SA1715	*gat*B	aspartyl/glutamyl-tRNA amidotransferase subunit B	0	30	<0.01
Translation factors	SA1100	*tsf*	elongation factor Ts	0	44	<0.01
Translation factors	SA1112	*infB*	translation initiation factor IF-2	0	20	<0.01
Protein folding	SA1410	*grp*E	heat shock protein GrpE	0	189	<0.01
**Cell envelope**						
Lipids	SA1357	*acc*C	acetyl-CoA carboxylase biotin carboxylase subunit	0	31	<0.01
Lipids	SA1523	*acc*D	acetyl-CoA carboxylase subunit beta	0	53	<0.01
Phospholipids	SA1126	*pgs*A	phosphatidylglycerophosphate synthase	0	70	<0.01
Peptidoglycan biosynthesis	SA1283	*pbp2*	penicillin-binding protein 2	0	36	<0.01
Teichoic acid biosynthesis	SA0244	*tar*F	teichoic acid biosynthesis protein F, putative	0	35	<0.01
Teichoic acid biosynthesis	SA0247	*tar*L	conserved hypothetical protein	0	47	<0.01
Teichoic acid biosynthesis	SA0793	*dlt*A	D-alanine--poly(phosphoribitol) ligase subunit 1	0	59	<0.01
Teichoic acid biosynthesis	SA0795	*dlt*C	D-alanine--poly(phosphoribitol) ligase subunit 2	0	82	<0.01
Cell division	SA1539	*ezr*A	septation ring formation regulator EzrA	0	23	<0.01
Cell division	SA2498	*noc*	conserved hypothetical protein	0	51	<0.01
Cell envelope - other	SA1126	*ebp*S	elastin binding protein	0	28	<0.01
**Carbon metabolism**						
Glycolysis	SA0728	*pgk*	phosphoglycerate kinase	0	33	<0.01
Glycolysis	SA1177	*tkt*	Transketolase	0	24	<0.01
**Respiratory pathway**						
Isoprenoid/Mevalonate biosynthesis	SA0342	-	hypothetical protein	0	37	<0.01
Isoprenoid/Mevalonate biosynthesis	SA0548	*mva*D	mevalonate diphosphate decarboxylase	0	43	<0.01
**Nucleotides**						
Purine biosynthesis	SA2027	*adk*	adenylate kinase	0	113	<0.01
Purine metabolism	SA1460	*rel*A	GTP pyrophosphokinase	0	21	<0.01
**Cofactors**						
Acetyl CoA/CoA	SA1054	*coa*BC	phosphopantothenoylcysteine decarboxylase	0	66	<0.01
NAD biosynthesis	SA1729	*pnc*B	nicotinate phosphoribosyltransferase	0	31	<0.01
Fe-sulphate cluster	SA0776	*suf*S	aminotransferase, class V superfamily, putative	0	69	<0.01
Fe-sulphate cluster	SA1315	*fer*	ferredoxin	0	187	<0.01
**Unknown/other**						
Unknown/other	SA0943-1	*pdh*A	pyruvate dehydrogenase E1 component subunit alpha	0	146	<0.01
Unknown/other	SA0674	*ltaS*	conserved hypothetical protein	0	21	<0.01
Unknown/other	SA1293	-	conserved hypothetical protein	0	62	<0.01
Unknown/other	SA1501	-	conserved hypothetical protein	0	134	<0.01
Unknown/other	SA1375	-	conserved hypothetical protein	0	70	<0.01
Unknown/other	SAS044	*dmp*I	4-oxalocrotonate tautomerase	0	252	<0.01
**Virulence**						
Adhesin	SA2434	*manP*	PTS mannose transporter subunit IIABC	0	20	<0.01
Adhesin	SA2291	*fnb*A	fibronectin-binding protein A	0	14	<0.01
Adhesin	SA0519	*sdr*C	Ser-Asp rich fibrinogen-binding, bone sialoprotein-binding protein	0	14	<0.01

Adhesin	SA2462	*ica*C	intercellular adhesion protein C	0	40	<0.01
Adhesin	SA1312	*ebp*S	elastin binding protein	0	28	<0.01
Exoenzyme	SA0309	*geh*	glycerol ester hydrolase	0	22	<0.01
Exoenzyme	SA1631	*spl*A	serine protease SplA	0	60	<0.01
Immune Evasion	SA0022	*ads*A	multifunctional 2',3'-cyclic-nucleotide 2'-phosphodiesterase/5'-nucleotidase/3'-nucleotidase	0	20	<0.01
Immune Evasion	SA0145	*cap*B	capsular polysaccharide synthesis protein Cap5B	0	57	<0.01
Immune Evasion	SA0150	*cap*G	capsular polysaccharide synthesis protein Cap5G	0	39	<0.01
Immune Evasion	SA0155	*cap*L	capsular polysaccharide synthesis protein Cap5L	0	32	<0.01
Secretion Systems	SA0271	*esx*A	virulence factor EsxA	0	149	<0.01

1-R GSSA DAP-R vs 1-S GSSA DAP-S antisense up

Category	RefGenN315	Symbol	Product	RPKM 1-S	RPKM 1-R	q-value
**DNA metabolism**						
DNA replication	SA0353	*ssb*	Antisense: *ssb*	0	943	<0.01
DNA replication	SA1415	*hol*A	Antisense: *hol*A	0	934	<0.01
DNA replication	SA1415	*hol*A	Antisense: *hol*A	0	934	<0.01
**RNA metabolism**						
Basic transcription machinery	SA0500	*rpo*B	Antisense: *rpoB*	0	934	<0.01
Basic transcription machinery	SA0501	*rpo*C	Antisense: *rpo*C	0	934	<0.01
RNA modification	SA0940	*rnj*A	Antisense: SA0940	0	934	<0.01
RNA modification	SA0940	*rnj*A	Antisense: SA0940	0	2802	<0.01
RNA regulation	SA1329	*fur*	Antisense: SA1329	0	1868	<0.01
RNA regulation	SA1174	*lex*A	Antisense: *lex*A	0	934	<0.01
RNA regulation	SA1174	*lex*A	Antisense: *lex*A	0	829	<0.01
**Protein synthesis**						
Ribosomal proteins	SA0352	*rps*F	Antisense: *rps*F	0	998	<0.01
Ribosomal proteins	SA0354	*rps*R	Antisense: *rps*R	0	934	<0.01
Ribosomal proteins	SA0354	*rps*R	Antisense: *rps*R	0	934	<0.01
Ribosomal proteins	SA0496	*rpl*A	Antisense: *rpl*A	0	934	<0.01
Ribosomal proteins	SA1067	*rpm*B	Antisense: *rpm*B	0	934	<0.01
Ribosomal proteins	SA0459	*rpl*Y	Antisense: *rpl*Y	0	934	<0.01
Ribosomal proteins	SA0459	*rpl*Y	Antisense: *rpl*Y	0	3669	<0.01
tRNA synthetase	SA0475	*lys*S	Antisense: *lys*S	0	934	<0.01
tRNA synthetase	SA0475	*lys*S	Antisense: *lys*S	0	934	<0.01
tRNA synthetase	SA0855	*trp*S	Antisense: *trp*S	0	934	<0.01
tRNA synthetase	SA1394	*gly*S	Antisense: *gly*S	0	934	<0.01
tRNA synthetase	SA1446	*ala*S	Antisense: *ala*S	0	2802	<0.01
Protein folding	SA1409	*dna*K	Antisense: *dna*K	0	1613	<0.01
Protein folding	SA1409	*dna*K	Antisense: *dna*K	0	934	<0.01
Protein translocation	SA1893	*yid*C	Antisense: *yid*C	0	1868	<0.01
**Cell envelope**						
Lipids Biosynthesis	SA1357	*acc*C	Antisense: *acc*C	0	934	<0.01
Cell wall/amino sugar	SA0457	*glm*U	Antisense: *glm*U	0	934	<0.01
Cell wall/amino sugar	SA0457	*glm*U	Antisense: *glm*U	0	934	<0.01
Cell wall/amino sugar	SA1959	*glm*S	Antisense: *glm*S	0	934	<0.01
Cell wall/amino sugar	SA1959	*glm*S	Antisense: *glm*S	0	1868	<0.01
Cell wall/amino sugar	SA1959	*glm*S	Antisense: *glm*S	0	113	<0.01
Peptidoglycan biosynthesis	SA0693	*mur*B	Antisense: *mur*B	0	934	<0.01
Peptidoglycan biosynthesis	SA1283	*pbp*2	Antisense: *pbp*2	0	934	<0.01
Peptidoglycan biosynthesis	SA1708	*mur*T	Antisense: *mur*T	0	934	<0.01
Peptidoglycan biosynthesis	SA1887	*ddl*	Antisense: *ddl*	0	934	<0.01
Teichoic acid biosynthesis	SA0593	*tag*H	Antisense: tag*H*	0	934	<0.01
Teichoic acid biosynthesis	SA0702	*llm*	Antisense: *llm*	0	1868	<0.01
Teichoic acid biosynthesis	SA0793	*dlt*A	Antisense: *dlt*A	0	934	<0.01
Cell division	SA1032	*sep*F	Antisense: *sep*F	0	934	<0.01
Cell envelope modification	SA1193	*fmt*C	Antisense: *fmt*C	0	2745	<0.01
**Carbon metabolism**						
Glycolysis	SA0823	*pgi*	Antisense: *pgi*	0	934	<0.01
Glycolysis	SA1927	*fba*A	Antisense: *fba*A	0	1807	<0.01
Pentose phosphate	SA1336	*zwf*	Antisense: *zwf*	0	2740	<0.01
**Respiratory pathway**						
Isoprenoid/Mevalonate biosynthesis	SA2334	*mva*S	Antisense: *mva*S	0	1868	<0.01
**Cofactors**						
Fe-sulphate cluster	SA0774	*suf*C	Antisense: *suf*C	0	934	<0.01

Fe-sulphate cluster	SA0775	*suf*D	Antisense: *suf*D	0	934	<0.01
**Unknown/other**						
Unknown/other	SA1425	-	Antisense: SA1425	0	934	<0.01
Unknown/other	SA1187	*pls*Y	Antisense: *pls*Y	0	934	<0.01
Unknown/other	SA1426	-	Antisense: SA1426	0	934	<0.01
Unknown/other	SA0816	-	Antisense: SA0816	0	1868	<0.01
Unknown/other	SA1445	-	Antisense: SA1445	0	934	<0.01
**Virulome**						
Adherence	SA0742	*clf*A	Antisense: *clf*A	0	934	<0.01
Adherence	SA2423	*clf*B	Antisense: *clf*B	0	934	<0.01
Adherence	SA2423	*clf*B	Antisense: *clf*B	0	687	<0.01
Adherence	SA2423	*clf*B	Antisense: *clf*B	0	934	<0.01
Adherence	SA0519	*sdr*C	Antisense: *sdr*C	0	1053	<0.01
Adherence	SA2586-87	*ica*A-*ica* D	Antisense: *ica*A-*ica*D	0	934	<0.01

1-R GSSA DAP-R vs 1-S GSSA DAP-S coding down

**Category**	**RefGenN315**	**Symbol**	**Product**	**RPKM 1-S**	**RPKM 1-R**	**q-value**
**Protein synthesis**						
Ribosomal proteins	SA1084	*rpl*S	50S ribosomal protein L19	452	0	<0.01
Ribosomal proteins	SA2043	*rps*S	30S ribosomal protein S19	400	0	<0.01
Ribosomal proteins	SA2046	*rpl*D	50S ribosomal protein L4	227	0	<0.01
Ribosomal proteins	SA2047	*rpl*C	50S ribosomal protein L3	229	0	<0.01
tRNA synthetase	SA1506	*thr*S	threonyl-tRNA synthetase	85	0	<0.01
Translation factors	SA1359	*efp*	elongation factor P	266	0	<0.01
Protein folding	SA1836	*gro*EL	molecular chaperone GroEL	96	0	<0.01
**Cell envelope**						
Peptidoglycan biosynthesis	SA1708	*mur*T	UDP-N-acetylmuramate tripeptide synthetase, putative	247	0	<0.01
Peptidoglycan biosynthesis	SA2057	*femh*B	FmhB protein	117	0	<0.01
Cell division	SA1029	*fts*Z	cell division protein FtsZ	132	0	<0.01
Cell division	SA2500	*gid*A	tRNA uridine 5-carboxymethylaminomethyl modification protein GidA	90	0	<0.01
**Carbon metabolism**						
Glycolysis	SA0458	*prs*	ribose-phosphate pyrophosphokinase	154	0	<0.01
Glycolysis	SA0731	*eno*	phosphopyruvate hydratase	230	0	<0.01
Pentose phosphate	SA1342	*gnd*	6-phosphogluconate dehydrogenase	108	0	<0.01
Intermediary metabolism	SA1088	*suc*C	succinyl-CoA synthetase subunit beta	420	0	<0.01
**Respiratory pathway**						
Isoprenoid/Mevalonate biosynthesis	SA2334	*mva*S	3-hydroxy-3-methylglutaryl-CoA synthase	89	0	<0.01
Thioredoxin	SA0992	*trx*A	thioredoxin	923	0	<0.01
**Nucleotides**						
Purine biosynthesis	SA1052	*gmk*	guanylate kinase	276	0	<0.01
Purine/Pyrimidine biosynthesis	SA0686	*ndr*E	ribonucleotide-diphosphate reductase subunit alpha	67	0	<0.01
**Cofactors**						
Folate	SA0474	*folK*	2-amino-4-hydroxy-6-hydroxymethyldihydropteridine pyrophosphokinase	333	0	<0.01
NAD biosynthesis	SA1422	*nad*D	nicotinate (nicotinamide) nucleotide adenylyltransferase	296	0	<0.01
Fe-sulphate cluster	SA0777	*suf*U	conserved hypothetical protein	312	0	<0.01
**Unknown/other**						
Unknown/other	SA1735	*ppa*C	manganese-dependent inorganic pyrophosphatase	181	0	<0.01
Unknown/other	SA1187	*pls*Y	conserved hypothetical protein	244	0	<0.01
Unknown/other	SA1744	*pmt*D	conserved hypothetical protein	209	0	<0.01
Unknown/other	SA0003	-	conserved hypothetical protein	687	0	<0.01
Unknown/other	SA0878	-	conserved hypothetical protein	201	0	<0.01
Unknown/other	SA1399	*ybe*Y	conserved hypothetical protein	187	0	<0.01
Unknown/other	SA1445	-	conserved hypothetical protein	777	46	<0.01
**Virulome**						
Exoenzyme	SA2463	*lip*	triacylglycerol lipase	72	0	<0.01

1-R GSSA DAP-R vs 1-S GSSA DAP-S down

Category	RefGenN315	Symbol	Product	RPKM 1-S	RPKM 1-R	q-value
**RNA metabolism**						
RNA regulation	SA0856	*spx*A	Antisense: *spx*A	3382	0	<0.01
RNA regulation	SA1438	*greA*	Antisense: *gre*A	3382	0	<0.01
**Protein synthesis**						
tRNA synthetase	SA1036	*ileS*	Antisense: *ile*S	3382	0	<0.01
tRNA synthetase	SA1717	*gat*C	Antisense: *gat*C	3382	0	<0.01
Protein translocation	SA2028	*sec*Y	Antisense: *sec*Y	3382	0	<0.01
**Cell envelope**						
Cell wall/amino sugar	SA1959	*glm*S	Antisense: *glm*S	3382	0	<0.01
Cell wall/amino sugar	SA1959	*glm*S	Antisense: *glm*S	3382	0	<0.01
Cell division	SA1279	*gps*B	Antisense: *gps*B	281	0	<0.01
**Carbon metabolism**						
Glycolysis	SA1927	*fba*A	Antisense: *fba*A	3382	0	<0.01
**Respiratory pathway**						
Isoprenoid/Mevalonate biosynthesis	SA2136	*fni*	Antisense: *fni*	3382	0	<0.01
**Cofactors**						
Fe-sulphate cluster	SA0778	*suf*B	Antisense: *suf*B	3382	0	<0.01
**Unknown/other**						
Unknown/other	SA1735	*ppa*C	Antisense: *ppa*C	3382	0	<0.01
Unknown/other	SA2093	*ssa*A	Antisense: *ssa*A	3382	0	<0.01
**Virulome**						
Secretion Systems	SA0281	*esa*D	Antisense: *esa*D	3382	0	<0.01
Adherence	SA0519	*sdr*C	Antisense: *sdr*C	3382	0	<0.01

2-R hGISA DAP-R vs 2-S GSSA DAP-S coding up

Category	RefGenST-398	Symbol	Product	RPKM 2-S	RPKM 2-R	q-value
**DNA metabolism**						
DNA replication	SAPIG1738	*dna*I	primosomal protein DnaI	0	118	<0.01
DNA packaging and segregation	SAPIG1680	-	hypothetical protein	0	169	<0.01
**RNA metabolism**						
Basic transcription machinery	SAPIG1626	*sig*A	RNA polymerase sigma factor RpoD (Sigma-A) (Sigma-43)	0	88	<0.01
**Protein synthesis**						
Ribosomal proteins	SAPIG2297	*rps*C	ribosomal protein S3	0	97	<0.01
Ribosomal proteins	SAPIG1733	*rpl*T	ribosomal protein L20	0	2	<0.01
tRNA synthetase	SAPIG1811	*leuS*	leucyl-tRNA synthetase	0	34	<0.01
tRNA synthetase	SAPIG1994	*gat*A	glutamyl-tRNA(Gln) amidotransferase subunit A (Glu-ADTsubunit A)	0	59	<0.01
Translation factors	SAPIG1594	*efp*	translation elongation factor P	0	1	<0.01
Translation factors	SAPIG1259	*tsf*	translation elongation factor Ts	0	58	<0.01
Protein folding	SAPIG1645	*dna*J	chaperone protein DnaJ	0	2	<0.01
**Cell envelope**						
Lipids	SAPIG1232	*acp*P	acyl carrier protein	0	244	<0.01
Lipids	SAPIG2112	*acp*S	holo-(acyl-carrier-protein) synthase	0	1	<0.01
Peptidoglycan biosynthesis	SAPIG1146	*mur*I	glutamate racemase	0	72	<0.01
Peptidoglycan biosynthesis	SAPIG2142	*mur*A	UDP-N-acetylglucosamine 1-carboxyvinyltransferase	0	60	<0.01
Teichoic acid biosynthesis	SAPIG0270	*tar*L	teichoic acid biosynthesis protein B	0	35	<0.01
**Carbon metabolism**						
Glycolysis	SAPIG0566	*prs*A	ribose-phosphate pyrophosphokinase	0	92	<0.01
Regulation	SAPIG1080	*pts*H	phosphocarrier protein HPr (Histidine-containing protein)	0	449	<0.01
**Respiratory pathway**						
Isoprenoid/Mevalonate biosynthesis	SAPIG0434	-	acetyl-CoA acetyltransferase (Acetoacetyl-CoA thiolase)	0	73	<0.01
Isoprenoid/Mevalonate biosynthesis	SAPIG2595	*mva*A	hydroxymethylglutaryl-CoA reductase, degradative	0	60	<0.01

Isoprenoid/Mevalonate biosynthesis	SAPIG2596	*mva*S	hydroxymethylglutaryl-CoA synthase	0	103	<0.01
**Cofactor**						
Fe-sulphate cluster	SAPIG0901	*suf*B	FeS assembly protein SufB	0	55	<0.01
**Unknown/other**						
Unknown/other	SAPIG1156	-	Conserved hypothetical protein	0	2	<0.01
Unknown/other	SAPIG1364	*dmp*I	4-oxalocrotonate tautomerase	0	2	<0.01
**Virulence**						
Immune evasion	SAPIG0168	*capF*	capsular polysaccharide synthesis enzyme	5	99	<0.01

2-R hGISA DAP-R vs 2-S GSSA DAP-S antisense up

Category	RefGenST-398	Symbol	Product	RPKM 2-S	RPKM 2-R	q-value
**RNA metabolism**						
RNA regulation	SAPIG0993	*spx*A	Antisense: *spx*A	0	1370	<0.01
**Protein synthesis**						
Ribosomal proteins	SAPIG1772	*rps*D	Antisense: *rps*D	0	2254	<0.01
Ribosomal proteins	SAPIG1772	*rps*D	Antisense: *rps*D	0	1027	<0.01
Translation factors	SAPIG0622	*fus*A	Antisense: *fus*A	0	3306	<0.01
Translation factors	SAPIG1696	*dtd*	Antisense: *dtd*	0	2204	<0.01
**Cell envelope**						
Cell envelope - other	SAPIG1361	*mpr*F	Antisense: *mpr*F	0	2052	<0.01
Cell envelope - other	SAPIG1361	*mpr*F	Antisense: *mpr*F	0	2204	<0.01
Cell envelope - other	SAPIG1361	*mpr*F	Antisense: *mpr*F	0	2743	<0.01
**Virulome**						
Adherence	SAPIG1050	*atl*	Antisense: *atl*	0	2204	<0.01
Adherence	SAPIG0637	*sdr*D	Antisense: *sdrD*	0	1102	<0.01
Immune evasion	SAPIG0165	*cap*C	Antisense: *cap*C	0	1102	<0.01

2-R hGISA DAP-R vs 2-S GSSA DAP-S coding down

Category	RefGenST-398	Symbol	Product	RPKM 2-S	RPKM 2-R	q-value
**DNA metabolism**						
DNA replication	SAPIG1627	*dna*G	DNA primase	64	0	<0.01
DNA replication	SAPIG1652	*hol*A	DNA polymerase III, delta subunit	68	0	<0.01
DNA packaging and segregation	SAPIG1471	*hu*	DNA-binding protein HU 1 (DNA-binding protein II) (HB)	134	0	<0.01
**RNA metabolism**						
RNA modification	SAPIG1086	*rnj*A	Conserved hypothetical protein	1	0	<0.01
RNA modification	SAPIG1239	*rim*M	16S rRNA processing protein RimM	1	0	<0.01
RNA modification	SAPIG1240	*trm*D	tRNA (guanine-N1)-methyltransferase	1	0	<0.01
**Protein synthesis**						
Ribosomal proteins	SAPIG1124	*rpm*F	ribosomal protein L32	218	0	<0.01
Ribosomal proteins	SAPIG1708	*rpm*A	ribosomal protein L27	17	3	<0.01
tRNA synthetase	SAPIG0009	*ser*S	seryl-tRNA synthetase	33	0	<0.01
tRNA synthetase	SAPIG1993	*gat*B	aspartyl/glutamyl-tRNA(Asn/Gln) amidotransferase subunit B (Asp/Glu-ADT subunit B)	19	0	<0.01
Protein folding	SAPIG1647	*grp*E	co-chaperone GrpE	251	0	<0.01
Protein folding	SAPIG2066	*gro*L	chaperonin GroL	28	0	<0.01
Protein translocation	SAPIG0948	*lep*B	signal peptidase I	84	0	<0.01
**Cell envelope**						
Cell wall/amino sugar	SAPIG2216	*glm*M	phosphoglucosamine mutase	26	0	<0.01
Peptidoglycan biosynthesis	SAPIG1695	*lyt*H	N-acetylmuramoyl-L-alanine amidase	1	0	<0.01
**Carbon metabolism**						
Glycolysis	SAPIG0945	*pgi*	glucose-6-phosphate isomerase	101	0	<0.01
**Respiratory pathway**						
Isoprenoid/Mevalonate biosynthesis	SAPIG0664	*mva*K1	mevalonate kinase	39	0	<0.01
**Cofactors**						
Folate	SAPIG0641	*fol*E2	conserved hypothetical protein	50	0	<0.01

Folate	SAPIG1717	*fol*C	folylpolyglutamate synthase (folylpoly-gamma-glutamatesynthetase) (fpgs) (tetrahydrofolate synthase)(tetrahydrofolylpolyglutamate synthase)	32	0	<0.01
Fe-sulphate cluster	SAPIG0897	*suf*C	FeS assembly ATPase SufC	46	0	<0.01
Fe-sulphate cluster	SAPIG1686	*isc*S	cysteine desulfurase	30	0	<0.01
**Unknown/other**						
Unknown/other	SAPIG2014	*ppa*C	manganese-dependent inorganic pyrophosphatase (Pyrophosphate phospho-hydrolase) (PPase)	31	0	<0.01
Unknown/other	SAPIG1270	*rpl*GA	ribosomal protein L7AE family	7	1	<0.01
Unknown/other	SAPIG1610	-	metallo-beta-lactamase superfamily protein	1	0	<0.01
Unknown/other	SAPIG1681	-	protein Stu1959	3	0	<0.01
**Virulome**						
Exotoxin	SAPIG1158	*hly/hla*	alpha-hemolysin (Alpha-toxin) (Alpha-HL)	71	0	<0.01
Exoenzyme	SAPIG2721	*lip*	lipase 2 (Glycerol ester hydrolase 2)	49	0	<0.01
Immune evasion	SAPIG0171	*cap*I	capsular polysaccharide biosynthesis protein Cap5I	7	0	<0.01

2-R hGISA DAP-R vs 2-S GSSA DAP-S antisense down

Category	RefGenST-398	Symbol	Product	RPKM 2-S	RPKM 2-R	q-value
**DNA metabolism**						
DNA replication	SAPIG0446	*ssb*	Antisense: *ssb*	1687	0	<0.01
DNA replication	SAPIG0446	*ssb*	Antisense: *ssb*	704	0	<0.01
DNA replication	SAPIG1652	*hol*A	Antisense: *hol*A	656	0	<0.01
DNA replication	SAPIG1999	*pcr*A	Antisense: pcr*A*	1898	0	<0.01
**RNA metabolism**						
Basic transcription machinery	SAPIG0617	*rpo*B	Antisense: *rpo*B	1898	0	<0.01
RNA modification	SAPIG1086	*rnj*A	Antisense: *rnj*A	3669	0	<0.01
RNA modification	SAPIG1086	*rnj*A	Antisense: *rnj*A	1898	0	<0.01
RNA regulation	SAPIG1564	*fur*	Antisense: *fur*	582	0	<0.01
**Protein synthesis**						
Ribosomal proteins	SAPIG1772	*rps*D	Antisense: *rps*D	346	0	<0.01
**Respiratory pathway**						
Isoprenoid/Mevalonate biosynthesis	SAPIG2596	*mva*S	Antisense: *mva*S	1265	0	<0.01
**Cofactor**						
Fe-sulphate cluster	SAPIG0901	*suf*B	Antisense: *suf*B	1581	0	<0.01
**Other/unknown**						
Other/unknown	SAPIG0796	*Ita*S	Antisense: *Ita*S	1898	0	<0.01
**Virulome**						
Adherence	SAPIG0636	*sdr*C	Antisense: *sdr*C	994	0	<0.01

3-R GISA DAP-R vs 3-S GSSA DAP-S coding up

Category	RefGenMW2	Symbol	Product	RPKM 3-S	RPKM 3-R	q-value
**DNA metabolism**						
DNA replication	MW1095	*pri*A	primosomal protein	0	13	<0.01
DNA replication	MW1147	*pol*C	DNA polymerase III PolC	0	9	<0.01
DNA replication	MW1514	*dna*G	DNA primase	0	47	<0.01
DNA packaging and segregation	MW0005	*gyr*B	DNA gyrase subunit B	0	42	<0.01
DNA packaging and segregation	MW1362	*hu*	DNA-binding protein II	0	159	<0.01
**RNA metabolism**						
RNA modification	MW1158	*rnj*B	Conserved hypothetical protein	0	54	<0.01
RNA modification	MW2630	*trm*E	tRNA modification GTPase TrmE	0	20	<0.01
RNA regulation	MW1149	*nus*A	transcription elongation factor NusA	0	35	<0.01
RNA regulation	MW0742	*rnr*	ribonuclease R	4	42	<0.01
RNA regulation	MW1249	*msr*R	peptide methionine sulfoxide reductase regulator MsrR	0	67	<0.01
**Protein synthesis**						
Ribosomal proteins	MW0501	*rps*G	30S ribosomal protein S7	0	70	<0.01
Ribosomal proteins	MW1010	*rpm*F	50S ribosomal protein L32	0	259	<0.01
Ribosomal proteins	MW1527	*rps*U	30S ribosomal protein S21	0	344	<0.01
Ribosomal proteins	MW2145	*rps*M	30S ribosomal protein S13	0	79	<0.01
tRNA synthetase	MW1580	*asp*S	aspartyl-tRNA synthetase	0	44	<0.01

tRNA synthetase	MW1607	*val*S	valyl-tRNA synthetase	0	24	<0.01
Translation factors	MW1140	*tsf*	elongation factor Ts	0	81	<0.01
Translation factors	MW1152	*inf*B	translation initiation factor IF-2	0	27	<0.01
Protein folding	MW1531	*dna*J	chaperone protein DnaJ	0	47	<0.01
**Cell envelope**						
Lipids	MW1114	*fab*G	3-oxoacyl- reductase (acyl-carrier protein)	0	50	<0.01
Lipids	MW1346	*bir*A	BirA bifunctional protein, putative	0	36	<0.01
Lipids	MW1479	*acc*C	acetyl-CoA carboxylase biotin carboxylase subunit	13	98	<0.01
Peptidoglycan biosynthesis	MW1143	*upp*S	undecaprenyl pyrophosphate synthase	0	41	<0.01
Peptidoglycan biosynthesis	MW2005	*mur*F	UDP-N-acetylmuramoylalanyl-D-glutamyl-2, 6-diaminopimelate-D-alanyl-D-alanyl ligase	0	36	<0.01
Teichoic acid biosynthesis	MW0814	*dlt*A	D-alanine--poly(phosphoribitol) ligase subunit 1	0	83	<0.01
Teichoic acid biosynthesis	MW0815	*dlt*B	DltB membrane protein	0	26	<0.01
Cell envelope - other	MW1247	*fmt*C/*mpr*F	oxacillin resistance-related FmtC protein	0	23	<0.01
**Carbon metabolism**						
Glycolysis	MW1642	*pfkA*	6-phosphofructokinase	0	118	<0.01
**Respiratory pathway**						
Menaquinone biosynthesis	MW0925	*men*A	1,4-dihydroxy-2-naphthoate octaprenyltransferase	0	33	<0.01
Thioredoxin	MW0726	*trx*B	thioredoxine reductase	39	226	<0.01
Thioredoxin	MW1028	*trx*A	thioredoxin	0	134	<0.01
**Nucleotides**						
Purine biosynthesis	MW2148	*adk*	adenylate kinase	0	75	<0.01
**Cofactors**						
Fe-sulphate cluster	MW0796	*suf*D	conserved hypothetical protein	0	24	<0.01
Fe-sulphate cluster	MW0798	*suf*U	conserved hypothetical protein	0	63	<0.01
Fe-sulphate cluster	MW1372	*fer*	ferredoxin	0	145	<0.01
**Other/unknown**						
Other/unknown	MW1240	*pls*Y	hypothetical protein	0	76	<0.01
Other/unknown	MW0837	-	hypothetical protein	0	143	<0.01
Other/unknown	MW0902	-	hypothetical protein	0	77	<0.01
**Virulome**						
Immune evasion	MW2341	*sbi*	IgG-binding protein SBI	0	24	<0.01
Immune evasion	MW0137	*cap*8N	capsular polysaccharide synthesis enzyme Cap8N	0	1	<0.01

3-R GISA DAP-R vs 3-S GSSA DAP-S antisense up

Category	RefGenMW2	Symbol	Product	RPKM 3-S	RPKM 3-R	q-value
**DNA metabolism**						
DNA replication	MW0342	*ssb*	Antisense: *ssb*	0	1442	<0.01
DNA packaging and segregation	MW1339	*recU*	Antisense: *rec*U	0	982	<0.01
**RNA metabolism**						
Basic transcription machinery	MW0497	*rpo*B	Antisense: *rpo*B	0	1561	<0.01
Basic transcription machinery	MW0497	*rpo*B	Antisense: *rpo*B	0	1099	<0.01
Basic transcription machinery	MW0498	*rpo*C	Antisense: *rpo*C	0	2114	<0.01
RNA modification	MW0972	*rnj*A	Antisense: *rnj*A	0	634	<0.01
RNA regulation	MW1452	*fur*	Antisense: *fur*	0	2464	<0.01
RNA regulation	MW1249	*msr*R	Antisense: *msr*R	0	3847	<0.01
RNA regulation	MW1249	*msr*R	Antisense: *msr*R	0	939	<0.01
**Protein synthesis**						
Ribosomal proteins	MW0500	*rps*L	Antisense: *rps*L	0	1099	<0.01
tRNA synthetase	MW1607	*val*S	Antisense: *val*S	0	1755	<0.01
**Cell envelope**						
Teichoic acid biosynthesis	MW0814	*dlt*A	Antisense: *dlt*A	0	927	<0.01
Cell division	MW1063	*fts*L	Antisense: *fts*L	0	1649	<0.01
**Respiratory pathway**						
Menaquinone biosynthesis	MW0925	*men*A	Antisense: *men*A	0	1099	<0.01
**Nucleotides**						
Purine/Pyrimidine biosynthesis	MW0692	*nrd*I	Antisense: *nrd*I	0	1099	<0.01
Purine/Pyrimidine biosynthesis	MW2051	*pyr*G	Antisense: *pyr*G	0	809	<0.01
**Cofactors**						
Folate	MW1606	*fol*C	Antisense: *fol*C	0	1099	<0.01

Fe-sulphate cluster	MW0797	*suf*S	Antisense: *suf*S	0	2143	<0.01
**Other/unknown**						
Other/unknown	MW1860	*ppa*C	Antisense: *ppa*C	0	1169	<0.01
Other/unknown	MW1240	*pls*Y	Antisense: *pls*Y	0	1596	<0.01
Other/unknown	MW1872	*pmt*C	Antisense: *pmt*C	0	1099	<0.01
Other/unknown	MW0544	*lip*L	Antisense: *lip*L	0	1420	<0.01
Other/unknown	MW1009	-	Antisense: MW1009	0	911	<0.01
Other/unknown	MW1692	-	Antisense: MW1692	0	455	<0.01
Other/unknown	MW2353	-	Antisense: MW2353	0	850	<0.01
**Virulome**						
Adherence	MW1956	*sdr*H	Antisense: *sdr*H	0	467	<0.01
Adherence	MW1956	*sdr*H	Antisense: *sdr*H	0	348	<0.01
Exoenzyme	MW1753	*spl*C	Antisense: *spl*C	0	3384	<0.01

3-R GISA DAP-R vs 3-S GSSA DAP-S coding down

**Category**	**RefGenMW2**	**Symbol**	**Product**	**RPKM 3-S**	**RPKM 3-R**	**q-value**
**DNA metabolism**						
DNA replication	MW1538	*hol*A	DNA polymerase III subunit delta	17	0	<0.01
DNA replication	MW1627	*dna*I	primosomal protein DnaI	23	0	<0.01
DNA replication	MW1628	*dna*B	chromosome replication initiation/membrane attachment protein	11	0	<0.01
DNA replication	MW1846	*pcr*A	ATP-dependend DNA helicase	8	0	<0.01
**RNA metabolism**						
RNA regulation	MW0018	*vic*R	response regulator	27	0	<0.01
RNA regulation	MW0019	*vic*K	two-component sensor histidine kinase	23	0	<0.01
**Protein synthesis**						
Ribosomal proteins	MW0341	*rps*F	30S ribosomal protein S6	79	0	<0.01
Ribosomal proteins	MW1139	*rps*B	30S ribosomal protein S2	60	0	<0.01
Ribosomal proteins	MW1597	*rpl*U	50S ribosomal protein L21	128	0	<0.01
Ribosomal proteins	MW2137	*rpl*M	50S ribosomal protein L13	52	0	<0.01
Ribosomal proteins	MW2154	*rpl*F	50S ribosomal protein L6	83	0	<0.01
Ribosomal proteins	MW2163	*rps*C	30S ribosomal protein S3	24	0	<0.01
Ribosomal proteins	MW2164	*rpl*V	50S ribosomal protein L22	189	0	<0.01
Ribosomal proteins	MW2165	*rps*S	30S ribosomal protein S19	141	0	<0.01
tRNA synthetase	MW0445	*met*S	methionyl-tRNA synthetase	10	0	<0.01
tRNA synthetase	MW0472	*lys*S	lysyl-tRNA synthetase	11	0	<0.01
tRNA synthetase	MW1626	*thr*S	threonyl-tRNA synthetase	12	0	<0.01
Translation factors	MW1583	*dtd*	D-tyrosyl-tRNA(Tyr) deacylase	53	0	<0.01
Protein folding	MW1533	*grp*E	heat shock protein GrpE	342	64	<0.01
Protein translocation	MW1118	*fts*Y	signal recognition particle	12	0	<0.01
**Cell envelope**						
Phospholipids	MW1669	*pls*C	1-acyl-sn-glycerol-3-phosphate acyltransferases domain protein	38	0	<0.01
Peptidoglycan biosynthesis	MW1033	*mur*I	glutamate racemase	27	0	<0.01
Peptidoglycan biosynthesis	MW2006	*ddl*	D-alanyl-alanine synthetase A	17	0	<0.01
Teichoic acid biosynthesis	MW0817	*dlt*D	poly D-alanine transfer protein (glycerophosphate chain)	18	0	<0.01
**Carbon metabolism**						
Glycolysis	MW0735	*pgk*	phosphoglycerate kinase	36	0	<0.01
Glycolysis	MW0736	*tpi*A	triosephosphate isomerase	32	0	<0.01
Glycolysis	MW0844	*pgi*	glucose-6-phosphate isomerase	14	0	<0.01
Glycolysis	MW0734	*gap*A	glyceraldehyde-3-phosphate dehydrogenase	19	0	<0.01
Pentose phosphate	MW1464	*gnd*	6-phosphogluconate dehydrogenase	17	0	<0.01
Regulation	MW0965	*pts*H	phosphocarrier protein HPr	125	0	<0.01
**Nucleotides**						
Pyrimidine	MW1141	*pyr*H	uridylate kinase	23	0	<0.01
**Cofactors**						
Folate	MW0470	*fol*B	dihydroneopterin aldolase	51	0	<0.01
Folate	MW0521	*fol*E2	putative GTP cyclohydrolase	29	0	<0.01
NAD biosynthesis	MW0888	*ppn*K	inorganic polyphosphate/ATP-NAD kinase	45	0	<0.01
SAM	MW1728	*met*K	S-adenosylmethionine synthetase	19	0	<0.01
**Other/unknown**						
GTP-binding protein	MW1548	-	GTP-binding protein YqeH	19	0	<0.01
Other/unknown	MW1973	*gcp*	putative DNA-binding/iron metalloprotein/AP endonuclease	22	0	<0.01
Other/unknown	MW0014	*gdp*P	hypothetical protein	10	0	<0.01
Other/unknown	MW0708	*gdp*S	hypothetical protein	16	0	<0.01

Other/unknown	MW1975	*tsa*B	hypothetical protein	39	0	<0.01
Other/unknown	MW2090	*dac*A	hypothetical protein	26	0	<0.01
Other/unknown	MW1106	*thi*N	hypothetical protein	78	0	<0.01
Other/unknown	MW1151	*rpl*GA	hypothetical protein	143	0	<0.01
Other/unknown	MW2218	-	hypothetical protein	436	0	<0.01
**Virulome**						
Adherence	MW0936	*atl*	autolysin	20	9	<0.01
Adherence	MW1880	*eap/map*	truncated cell surface protein map-w	11	0	<0.01
Adherence	MW0516	*sdr*C	Ser-Asp rich fibrinogen-binding bone sialoprotein-binding protein	22	0	<0.01
Adherence	MW0517	*sdr*D	Ser-Asp rich fibrinogen-binding bone sialoprotein-binding protein	11	0	<0.01
Immune evasion	MW0259	*esa*A	Type VII secretion protein Esa	17	0	<0.01
Immune evasion	MW0139	*cap*8P	capsular polysaccharide synthesis enzyme Cap8P	14	0	<0.01
Exotoxin	MW1044	*hly/hla*	Alpha-hemolysin	21	0	<0.01
Exotoxin	MW1959	*hld*	delta-hemolysin	1865	0	<0.01
Exotoxin	MW2343	*hlg*C	gamma-hemolysin component C	38	0	<0.01
Exotoxin	MW0051	*seh*	enterotoxin H	28	0	<0.01
Exotoxin	MW2344	*hlg*B	gamma-hemolysin component B	21	0	<0.01
Membrane-acting toxin and Superantigen	MW0084	*spa*	immunoglobulin G binding protein A precursor	23	0	<0.01
Exoenzymes	MW1754	*spl*B	serine protease SplB	28	0	<0.01
Biofilm	MW2586	*ica*A	N-glycosyltransferase	13	0	<0.01

3-R GISA DAP-R vs 3-S GSSA DAP-S antisense down

Category	RefGenMW2	Symbol	Product	RPKM 3-S	RPKM 3-R	q-value
**DNA metabolism**						
DNA replication	MW0001	*dna*A	Antisense: *dna*A	2831	0	<0.01
DNA replication	MW0002	*dna*N	Antisense: *dna*N	1522	0	<0.01
DNA replication	MW0002	*dna*N	Antisense: *dna*N	1522	0	<0.01
DNA replication	MW0342	*ssb*	Antisense: *ssb*	761	0	<0.01
DNA replication	MW0342	*ssb*	Antisense: *ssb*	761	0	<0.01
DNA packaging and segregation	MW0005	*gyr*B	Antisense: *gyr*B	99	0	<0.01
DNA packaging and segregation	MW0006	*gyr*A	Antisense: *gyr*A	99	0	<0.01
DNA packaging and segregation	MW0006	*gyr*A	Antisense: *gyr*A	99	0	<0.01
DNA packaging and segregation	MW1133	*top*A	Antisense: *top*A	761	0	<0.01
**RNA metabolism**						
RNA modification	MW0972	*rnj*A	Antisense: *rnj*A	1381	0	<0.01
Basic transcription machinery	MW0497	*rpo*B	Antisense: *rpo*B	1522	0	<0.01
**Protein synthesis**						
Ribosomal proteins	MW1010	*rpm*F	Antisense: *rpm*F	761	0	<0.01
Ribosomal proteins	MW1139	*rps*B	Antisense: *rps*B	761	0	<0.01
Ribosomal proteins	MW1139	*rps*B	Antisense: *rps*B	761	0	<0.01
Ribosomal proteins	MW1662	*rps*D	Antisense: *rps*D	761	0	<0.01
Ribosomal proteins	MW2150	*rpl*O	Antisense: *rpl*O	761	0	<0.01
Ribosomal proteins	MW2159	*rpl*N	Antisense: *rpl*N	761	0	<0.01
Ribosomal proteins	MW2161	*rpm*C	Antisense: *rpm*C	761	0	<0.01
Ribosomal proteins	MW2165	*rps*S	Antisense: *rps*S	761	0	<0.01
tRNA synthetase	MW1517	*gly*S	Antisense: *gly*S	761	0	<0.01
tRNA synthetase	MW1568	*ala*S	Antisense: *ala*S	761	0	<0.01
tRNA synthetase	MW1626	*thr*S	Antisense: *thr*S	761	0	<0.01
Translation factors	MW1152	*inf*B	Antisense: *inf*B	761	0	<0.01
Translation factors	MW1152	*inf*B	Antisense: *inf*B	761	0	<0.01
Translation factors	MW0743	*smp*B/*ssr*P	Antisense: *smp*B/*ssr*P	1522	0	<0.01
Protein folding	MW1532	*dna*K	Antisense: *dna*K	761	0	<0.01
Protein folding	MW1532	*dna*K	Antisense: *dna*K	761	0	<0.01
Protein folding	MW1953	*gro*EL	Antisense: *gro*EL	761	0	<0.01
Protein translocation	MW1587	*sec*F	Antisense: *sec*F	1522	0	<0.01
**Cell envelope**						
Lipids	MW2023	*fab*Z	Antisense: *fab*Z	761	0	<0.01
Cell wall/amino sugar	MW0454	*glm*U	Antisense: *glm*U	761	0	<0.01
Peptidoglycan biosynthesis	MW1833	*mur*T	Antisense: *mur*T	761	0	<0.01
Peptidoglycan biosynthesis	MW1833	*mur*T	Antisense: *mur*T	761	0	<0.01
Peptidoglycan biosynthesis	MW2005	*mur*F	Antisense: *mur*F	761	0	<0.01
Peptidoglycan biosynthesis	MW2006	*ddl*	Antisense: *ddl*	1522	0	<0.01
Cell division	MW1068	*fts*A	Antisense: *fts*A	761	0	<0.01
Cell envelope - other	MW1247	*fmt*C/*mpr*F	Antisense: *fmt*C/*mpr*F	761	0	<0.01
Cell envelope - other	MW1247	*fmt*C/*mpr*F	Antisense: *fmt*C/*mpr*F	1522	0	<0.01

**Carbon metabolism**						
Glycolysis	MW0738	*eno*	Antisense: *eno*	761	0	<0.01
Glycolysis	MW1641	*pyk*A	Antisense: *pyk*A	761	0	<0.01
Glycolysis	MW2049	*fba*A	Antisense: *fba*A	761	0	<0.01
**Respiratory pathway**						
Isoprenoid/Mevalonate biosynthesis	MW0231	*isp*D/ *tar*I	Antisense: *isp*D/ *tar*I	761	0	<0.01
**Nucleotides**						
Purine/Pyrimidine biosynthesis	MW1092	*gm*K	Antisense: *gm*K	761	0	<0.01
Purine/Pyrimidine biosynthesis	MW0693	*nrd*E	Antisense: *nrd*E	1522	0	<0.01
**Cofactors**						
Acetyl CoA/CoA	MW2054	*coa*A	Antisense: *coaA*	761	0	<0.01
Folate	MW1316	*dfr*A	Antisense: *dfr*A	761	0	<0.01
Folate	MW1606	*fol*C	Antisense: *fol*C	761	0	<0.01
Folate	MW1606	*fol*C	Antisense: *fol*C	1488	0	<0.01
NAD biosynthesis	MW1545	*nad*D	Antisense: *nadD*	1522	0	<0.01
NAD biosynthesis	MW1545	*nad*D	Antisense: *nadD*	761	0	<0.01
SAM	MW1728	*met*K	Antisense: *met*K	761	0	<0.01
Fe-sulphate cluster	MW0797	*suf*S	Antisense: *suf*S	761	0	<0.01
Fe-sulphate cluster	MW0798	*suf*U	Antisense: *suf*U	323	0	<0.01
**Other/unknown**						
GTP binding	MW1594	*obg*E	Antisense: *obg*E	761	0	<0.01
GTP binding	MW1364	*eng*A	Antisense: *eng*A	1522	0	<0.01
Other/unknown	MW1860	*ppa*C	Antisense: *ppa*C	761	0	<0.01
Other/unknown	MW0681	*ltaS*	Antisense: *ltaS*	761	0	<0.01
Other/unknown	MW1104	*rsg*A	Antisense: *rsg*A	761	0	<0.01
Other/unknown	MW1350	-	Antisense: MW1350	1522	0	<0.01
Other/unknown	MW1727	*smd*A	Antisense: *smd*A	761	0	<0.01
Other/unknown	MW0544	*lip*L	Antisense: *lip*L	761	0	<0.01
Other/unknown	MW0837	-	hypothetical protein	761	0	<0.01
**Virulome**						
Adherence	MW0936	*atl*	Antisense: *atl*	761	0	<0.01
Adherence	MW0764	*clf*A	Antisense: *clf*A	1522	0	<0.01
Adherence	MW0764	*clf*A	Antisense: *clf*A	761	0	<0.01
Adherence	MW0764	*clf*A	Antisense: *clf*A	282	44	<0.01
Adherence	MW0764	*clf*A	Antisense: *clf*A	761	0	<0.01
Adherence	MW2551	*clf*B	Antisense: *clf*B	249	54	<0.01
Adherence	MW2562	*cna*	Antisense: *cna*	761	0	<0.01
Adherence	MW0517	*sdr*D	Antisense: *sdr*D	680	0	<0.01
Effector delivery system	MW0263	*ess*C	Antisense: *ess*C	1522	0	<0.01
Exotoxin	MW2342	*hlg*A	Antisense: *hlg*A	761	0	<0.01
Exoenzymes	MW2590	*lip*	Antisense: *lip*	761	0	<0.01
Exoenzymes	MW0932	*ssp*A	Antisense: *ssp*A	761	0	<0.01
Exoenzymes	MW1754	*spl*B	Antisense: *spl*B	28	0	<0.01
Membrane-acting toxin and Superantigen	MW0084	*spa*	Antisense: *spa*	761	0	<0.01
Membrane-acting toxin and Superantigen	MW0084	*spa*	Antisense: *spa*	761	0	<0.01
Immune evasion	MW2341	*sbi*	Antisense: *sbi*	1522	0	<0.01
Immune evasion	MW0138	*cap*8O	Antisense: *cap*8O	761	0	<0.01
