# Supplementary material for: Mutation- and Transcription-Driven Omic Burden of Daptomycin/Dalbavancin-R and Glycopeptide-RS Fitness Costs in High-Risk MRSA: A Nexus in Antimicrobial Resistance Mechanisms—Genome Proneness—Compensatory Adaptations
Source: Antibiotics (Basel). 2025 May 2;14(5):465. doi: 10.3390/antibiotics14050465 (PMC12108176; doi:10.3390/antibiotics14050465)
Supplement: Supplementary file 1 [file antibiotics-14-00465-s001.zip › Table S3.pdf]

Table S3. Genome position and Log2 Fold-Change in transcriptomics and real-time qPCR in all sample

dltA	Locus Tag RefGen	Product	RefGen Position (nt)	RNA size (bp)	Library	RPKM 1R	RPKM 1S	Log2 Fold-Change transcriptomics	Log2 Fold Change real-time qPCR
1-S/R HA-MRSA	SA0793	*dlt*A	898448-899905	1457	SI	59	0	≈9.96	≈9.87
		as*dlt*A	901117-901133	16	SI	934	0		
2-S/R LA-hGISA	SAPIG0915	*dlt*A	954574-956031	1457	SI	56	30	≈5.22	≈5.15
		as*dlt*A	955546-955527	19	SI	1102	0		
3-S/R CA-GISA	MW0814	*dlt*A	898360-899817	1457	SI	83	0	≈10.61	≈9.98
		as*dlt*A	898665-898634	31	SI	927	0		
		as*dlt*A	898828-898796	32	SI	549	0		
		as*dlt*A	900238-899145	1093	SI	10	0		

***mpr*F/*fmt*C**	Locus Tag RefGen	Product	RefGen Position (nt)	RNA size (bp)	Library	RPKM 1R	RPKM 1S	Log2 Fold-Change transcriptomics	Log2 Fold Change real-time qPCR
1-S/R HA-MRSA	SA1193	*fmt*C	1363612-1366134	2522	SI	0	0	≈11.43	≈11.30
		as*fmt*C	1366017-1365969	48	SI	2745	0		
2-S/R LA-hGISA	SAPIG1193	*fmt*C	1432138-1434660	2522	SI	26	30	≈3.78	≈3.25
		as*fmt*C	1432819-1432791	28	SI	2052	0		
		as*fmt*C	1433276-1433257	19	SI	1102	0		
		as*fmt*C	1433481-1433455	26	SI	2204	0		
		as*fmt*C	1433867-1433821	46	SI	2743	0		
		as*fmt*C	1434062-1434032	30	SI	0	632		
		as*fmt*C	1434362-1434338	24	SI	1102	0		
3-S/R CA-GISA	MW1247	*fmt*C	1365602-1368124	2522	SI	23	0	≈-2.39	≈-2.0
		as*fmt*C	1365760-1365737	23	SI	0	761		
		as*fmt*C	1365947-1365925	22	SI	549	761		
		as*fmt*C	1366276-1366249	27	SI	0	1522		

***mur*F**	Locus Tag RefGen	Product	RefGen Position (nt)	RNA size (bp)	Library	RPKM 1R	RPKM 1S	Log2 Fold-Change transcriptomics	Log2 Fold Change real-time qPCR
1-S/R HA-MRSA	SA1886	*mur*F	2139297-2137939	1358	SI	0	0	0	0
2-S/R LA-hGISA	SAPIG2123	*mur*F	2206060-2204705	1355	SI	0	18	≈-4.25	≈-3.81
3-S/R CA-GISA	MW2005	*mur*F	2166005-2164647	1358	SI	36	0	≈5.21	≈4.95

asRNA sucA	Locus Tag RefGen	Product	RefGen Position (nt)	RNA size (bp)	Library	RPKM 1R	RPKM 1S	Log2 Fold-Change transcriptomics	Log2 Fold Change real-time qPCR
1-S/R HA-MRSA	SA1245	-	-	-	-	-	-	-	-
2-S/R LA-hGISA	SAPIG1415	as*sucA*	1489805-1489833	28	SI	1786	0	≈10.80	≈10.73

3-S/R CA-GISA	MW1303	as*sucA*	1423626-1423663	37	SI	969	0	≈9.92	≈9.82

Legend:

- Log2 fold-change >0 over-expression of the R vs S
- Log2 fold-change <0 under-expression of the R vs S
- Log2 fold-change=0 no difference between R vs S
