# Supplementary material for: Mutation- and Transcription-Driven Omic Burden of Daptomycin/Dalbavancin-R and Glycopeptide-RS Fitness Costs in High-Risk MRSA: A Nexus in Antimicrobial Resistance Mechanisms—Genome Proneness—Compensatory Adaptations
Source: Antibiotics (Basel). 2025 May 2;14(5):465. doi: 10.3390/antibiotics14050465 (PMC12108176; doi:10.3390/antibiotics14050465)
Supplement: Supplementary file 1 [file antibiotics-14-00465-s001.zip › Table S4.pdf]

Table S4. List of acronyms in the text

AMR: antimicrobial resistance
CA-MRSA: community-acquired MRSA
DAL: dalbavancin
DAP: daptomycin
EGs: essential genes
FCs: fitness costs
GISA: glycopeptide-intermediate S. aureus
GLYs: glycopeptides
HA-MRSA: hospital-associated MRSA
hGISA: heterogeneous glycopeptide-intermediate S. aureus
LA-MRSA: livestock-associated MRSA
MOA: mechanism of action
RS: reduced susceptibility
TEC: teicoplanin
VAN: vancomycin
VGs: virulence genes
XDR: extremely drug resistance
